# Supplementary material for: Testing the effectiveness of a weight loss intervention to enhance self-regulation in adults who are obese: protocol for a randomised controlled trial
Source: BMJ Open. 2019 Dec 8;9(12):e031572. doi: 10.1136/bmjopen-2019-031572 (PMC6924834; doi:10.1136/bmjopen-2019-031572)
Supplement: Supplementary data [file bmjopen-2019-031572supp001.pdf]

List of 53 Weight Loss Actions

Category Red: Eating in a structured way
1. Plan all meals for the day in advance (what and when)
2. Eat no more than three times
3. Skip a meal
4. No calories after 8pm
5. Check the calorie count of everything you want to eat or drink
6. Set yourself a calorie goal and stick to it
7. Have a "fasting" day with less than 800kcal
8. Keep a diary of what you eat and how you feel
9. Check your portion size
10. Only eat when sitting at a table

Category Orange: Avoiding or swapping specific foods
1. Don't eat between meals
2. Cut out crisps, biscuits, cakes and sweets
3. Cut out fried food
4. Have only one course at meal-times
5. Cut out carbs
6. Swap unhealthy snacks for fruits and vegetables
7. Swap rice/potatoes/pasta for extra vegetables
8. Use meal replacement products
9. Swap unhealthy snacks with 6-8 individual nuts

Category Yellow: Changing what you drink
1. Drink only water or unsweetened coffee or tea
2. Swap sugary soft drinks with diet or no sugar versions
3. Do not drink alcohol
4. Drink a pint of water before each meal
5. Swap juices or smoothies with whole fruit and vegetables

Category Green: Creating a healthier diet
1. Eat at least 5 portions of fruit or vegetables each day
2. Snack only on vegetables
3. Eat only foods with a green nutrition label for total fat
4. Eat only foods with a green nutrition label for sugar
5. Make sure half of your main meal of the day is a salad or vegetables
6. Swap rice/potatoes/pasta with extra vegetables
7. Swap fatty meats with lean meats

Frie K, *et al. BMJ Open* 2019; 9:e031572. doi: 10.1136/bmjopen-2019-031572

Category Blue: Meal-time tactics
1. Eat slowly or 20 chews per bite
2. Focus on your food while eating
3. Stop eating before you feel full
4. Use smaller plates and bowls
5. Cut food into smaller pieces
6. Eat for less than 20 minutes at a time

Category Purple: Burn more calories
1. Walk up and down a flight of stairs for as long as you can
2. Go cycling for as long as you can
3. Go swimming for as long as you can
4. Stretching Exercises
5. Attend an exercise class
6. Play a group sport
7. Go to the gym
8. Exercise at home with the 21-minutes NHS Choices workout
9. Brisk walking for as long as you can

Category Pink: Be more active as part of your daily life
1. Walk 10,000 steps
2. Walk/cycle instead of taking the bus or car
3. Go for a walk with your friend(s)
4. Stand up while working
5. Take the stairs whenever you can
6. Have an active day with your family or friends
7. Stand up while watching TV

Frie K, *et al. BMJ Open* 2019; 9:e031572. doi: 10.1136/bmjopen-2019-031572
