# Supplementary material for: Testing the effectiveness of a weight loss intervention to enhance self-regulation in adults who are obese: protocol for a randomised controlled trial
Source: BMJ Open. 2019 Dec 8;9(12):e031572. doi: 10.1136/bmjopen-2019-031572 (PMC6924834; doi:10.1136/bmjopen-2019-031572)
Supplement: Supplementary data [file bmjopen-2019-031572supp002.pdf]

STATISTICAL ANALYSIS PLAN

Study Title:

A proof of concept trial of a self-regulation intervention for weight loss
(PREVAIL Trial)

**Chief Investigator:**	Kerstin Frie, Doctoral Candidate,<br><br>Nuffield Department of Primary Care Health Sciences
**Co-Investigators:**	Dr Jamie Hartmann-Boyce[1]<br><br>Prof. Susan Jebb[1]<br><br>Prof. Paul Aveyard[1]<br><br>1. Nuffield Department of Primary Care Health Sciences

Version History

Version:	Version Date:	Changes:
1.0	**21st October 2019**	

Frie K, *et al. BMJ Open* 2019; 9:e031572. doi: 10.1136/bmjopen-2019-031572

Table of Contents

INTRODUCTION **3**
PREFACE 3
PURPOSE AND SCOPE OF THE STATISTICAL ANALYSIS PLAN 3
BACKGROUND 3
TRIAL OVERVIEW 3
OBJECTIVES 4
PRIMARY OUTCOME 4
SECONDARY OUTCOMES (PROCESS EVALUATION) 4
TRIAL DESIGN **4**
OUTCOME MEASURES 4
PRIMARY OUTCOME 4
SECONDARY OUTCOMES (PROCESS EVALUATION) 5
BLINDING IN THE ANALYSIS STAGE 6
PRIMARY & SECONDARY ANALYSIS **6**
PRIMARY OUTCOME 6
SECONDARY OUTCOMES (PROCESS EVALUATION) 6
ANALYSIS – GENERAL CONSIDERATIONS **7**
DATA CLEANING 7
DESCRIPTIVE STATISTICS AND PARTICIPANT CHARACTERISTICS 7
DEFINITION OF POPULATION FOR ANALYSIS 7
HANDLING MISSING DATA 8
HANDLING OUTLIERS 8
MODEL ASSUMPTIONS 8
APPENDICES **9**

PREVAIL Trial Statistical Analysis Plan, Version 1.0, 21/10/2019

INTRODUCTION

PREFACE

The SAP supports the study protocol version 1.1, dated 07/12/2018. Quantitative analysis will be carried out using SPSS, version 24, and R Studio, version 1.0.153. Qualitative analysis will be run using NVivo, version 11.4.2.

PURPOSE AND SCOPE OF THE STATISTICAL ANALYSIS PLAN

The purpose of the plan is to set out the main analysis as stated in the protocol.

BACKGROUND

The effectiveness of self-weighing for weight loss has often been ascribed to an automatic self-regulation mechanism. It starts with the contextualisation of weight measurements, thus providing an opportunity to reflect on previous behaviours, and enabling the planning and performance of weight loss actions. A study employing self-weighing as a standalone intervention did not find a significant weight loss effect, raising the question whether self-regulation is performed naturally after weighing. We addressed this question in a think-aloud study, where twenty-four participants were asked to record their thoughts and feelings during daily weighing for eight weeks, without being prompted to self-regulate. On 90% of occasions, participants contextualised their weight measurements and on 58% participants reflected on previous behaviours. Only on 20% of occasions did participants plan actions. Specific action-planning, defining a concrete action and time plan, was rare (6%). The frequency of specific action-planning was, however, significantly predictive of weight loss. Hence, the study provided support to the notion that completing the last step of the self-regulation process can elicit weight loss. However, the think-aloud study also showed that self-regulation does not often occur autonomously, and that people need support in developing self-regulation skills, especially action-planning.

TRIAL OVERVIEW

With this study we aim to test the early effectiveness of an intervention guiding people through the iterative self-regulation process. One hundred participants with a BMI≥30 kg/m2 will be randomised to either the control or intervention group. The control group will be asked to weigh themselves daily for eight weeks, the intervention group will be encouraged to follow the self-regulation intervention. They will be prompted to weigh daily, track their weight using an app, plan daily actions for weight loss and reflect on their action plans on a weekly basis. This self-regulation cycle will allow them to experiment with different weight loss strategies and identify effective and sustainable actions. Primary and process outcomes will be measured at baseline and 8-weeks follow-up.

PREVAIL Trial Statistical Analysis Plan, Version 1.0, 21/10/2019

OBJECTIVES

Primary Outcome

The primary objective of this trial is to test the concept of whether an intervention, which trains individuals in self-regulatory processes following self-weighing, increases early weight loss in comparison to unsupported daily weighing.

Secondary Outcomes (Process Evaluation)

A mix of quantitative and qualitative measures will be used to undertake a process evaluation of the intervention and investigate moderators of effectiveness. This will include a quantitative assessment of participants' adherence to intervention components. The resulting measures will be used to assess whether adherence is predictive of weight change. Furthermore, as cognitive processing and reflection skills are required for self-regulation, the predictive value of the highest educational qualification achieved will be assessed for the outcome weight change. Since weighing is a major component of the treatment in both the control and intervention group, a potential link between liking of weighing at baseline and weight change at follow-up will also be investigated. Furthermore, the perceived usefulness of intervention features shall be explored quantitatively. The association between the overall intervention rating and weight change from baseline to follow-up will be assessed. Qualitative analysis of semi-structured interviews will help to evaluate acceptability and feasibility of the intervention, as well as identify barriers and unmet needs.

TRIAL DESIGN

An individually randomised, two arm, parallel group design, assessing superiority of the self-regulation intervention over daily self-weighing alone. Participation lasts eight weeks and participants are randomly allocated to the intervention or control condition on a 1:1 basis, stratified by GP practice. The intervention features are detailed in the study protocol. The study population includes 100 adults ≥18 years of age, with a BMI≥30 kg/m^2, who did not weigh themselves more than once a week at time of enrolment. For further details, refer to the trial protocol.

OUTCOME MEASURES

PRIMARY OUTCOME

Primary Objective	Measures	Timepoints
**Early effectiveness**	Change in weight from baseline to follow-up, using a digital scale.	Baseline to follow-up (8-9 weeks)

Frie K, *et al. BMJ Open* 2019; 9:e031572. doi: 10.1136/bmjopen-2019-031572

PREVAIL Trial Statistical Analysis Plan, Version 1.0, 21/10/2019

SECONDARY OUTCOMES (PROCESS EVALUATION)

Objectives	Outcome Measures	Timepoints of evaluation of this outcome measure
**1. Adherence**	Intervention Condition: Adherence to weight-tracking will be measured as the proportion of days for which a weight was recorded in the weight-tracking app. Adherence to action planning and reflection/evaluation will be measured by calculating the proportion of days/weeks on which the respective questionnaires were completed. The proportionate adherence to daily weighing will be pooled from weight-tracking and action planning questionnaire data. A composite adherence score will be calculated by averaging the adherence measures for daily weighing, weight-tracking, action planning and reflection/evaluation.<br>Control Condition: Adherence to daily weighing will be measured as the proportion of days for which a weight was recorded on the BodyTrace scales server or for which a written record was provided by the participant.	Throughout intervention period
**2. Baseline Questionnaire**	A baseline questionnaire measures demographic characteristics such age, gender, ethnicity, highest educational degree and employment status. The questionnaire further asks participants whether they like weighing themselves on a scale from 1 (dislike it a great deal) to 5 (like it a great deal).	At baseline
**3. Final Questionnaire**	A final questionnaire collects ratings on a scale from 1 (not useful) to 10 (very useful) for each intervention component, as well as for the intervention overall, from participants in the intervention condition.	At follow-up
**3. Semi-structured Interviews**	Semi-structured interviews are conducted by the researcher at follow-up in order to gain insights into the participants' opinions regarding the intervention components, as well as to collect feedback on acceptability and feasibility, alongside identifying barriers and unmet needs.	At follow-up

BLINDING IN THE ANALYSIS STAGE

It is not possible to blind the researcher delivering the intervention and analysing the trial data to treatment group due to the nature of the intervention. The primary outcome, change in weight, is measured objectively and is therefore unlikely to be biased. Adherence to self-regulation steps is also measured objectively through the frequency of weight logs and completed questionnaires in control and intervention group. The evaluations of treatment components are measured in an online questionnaire without the researchers input. Blinding of the researcher who conducts and analyses the semi-structured interviews with intervention group participants will not be possible.

PRIMARY & SECONDARY ANALYSIS

PRIMARY OUTCOME

The statistical analysis of the primary outcome, effectiveness of the intervention for weight loss, will be carried out both on the basis of intention-to-treat (ITT) and per-protocol (PP). For the ITT analysis, participants will be analysed according to their allocated intervention group. We will endeavour to obtain full follow-up data on every participant to allow full ITT analysis. Where we are unable to meet participants for follow-up, we will try to record self-reported weight at eight-week follow-up by telephone or email. For the PP analysis, we will exclude participants who stopped following their allocated intervention at some point throughout the study. In both cases, a linear mixed effects model, predicting weight at follow-up while adjusting for baseline weight (fixed effect) and GP practice (random effect), will assess the effect of condition (fixed effect). All analysis will be done at a 5% two-sided significance level.

SECONDARY OUTCOMES (PROCESS EVALUATION)

Means and standard deviations of adherence rates to the different intervention components will be calculated. Adherence rates for daily weighing in the control and intervention condition will be compared using independent samples t-tests. Means and standard deviations of final questionnaire ratings will be calculated.

Further linear mixed effects models will be calculated to assess different potential moderators of effectiveness.

Highest educational qualification: Since the majority of participants in the sample have a university degree or equivalent, and the rest are distributed in small quantities across the other qualification levels, a binary variable with 1=university degree or equivalent and 0=no university degree or equivalent will be calculated. A linear mixed effects model using the same parameters as in the primary analysis, and adding the binary variable educational qualification, as well as the interaction term education*condition, will be run in order to test

for an effect on weight change.

Adherence: We will replicate the linear mixed effects model of the primary analysis, and add the composite adherence score in the intervention group/the daily weighing adherence measure in the control condition in order to assess the predictive value of overall adherence for weight change. The model will also include an interaction term condition*adherence.

Liking of Weighing: A linear mixed effects model will test the predictive value of liking of weighing at baseline for weight change, adjusting for the same parameters as in the primary analysis. Liking of weighing will be added both as an independent parameter, as well as in an interaction term with condition.

Intervention Rating (only intervention condition): A linear mixed effects model will test the predictive value of the overall intervention rating for weight change, adjusting for both baseline weight and GP practice.

All of the above analyses will be done at a 5% two-sided significance level.

All interview audio-recordings will be transcribed and entered into the NVivo software package (QSR International) for qualitative data analysis. Framework analysis according to Ritchie and Spencer will assess the participant's experiences and perceptions of the different intervention components. The findings will be put into context with the results from the final questionnaire. Inductive thematic analysis following Braun and Clarke will explore additional themes, including acceptability, barriers and unmet needs.

ANALYSIS – GENERAL CONSIDERATIONS

DATA CLEANING

Prior to the final data lock, data cleaning will be performed, including checking that all appropriate data has been reported.

DESCRIPTIVE STATISTICS AND PARTICIPANT CHARACTERISTICS

A table will present the baseline characteristics by trial arm and overall (Appendix 1).

Continuous variables will be summarised using means and standard deviations. Categorical variables will be summarised using counts and percentages. Data will be analysed using R.

DEFINITION OF POPULATION FOR ANALYSIS

The statistical analysis of efficacy outcomes will be carried out on the basis of intention-to-treat (ITT). We will endeavour to obtain full follow-up data on every participant to allow full ITT analysis, but we will inevitably experience the problem of missing data due to withdrawal, loss to follow up, or non-response to questionnaire items.

Frie K, *et al. BMJ Open* 2019; 9:e031572. doi: 10.1136/bmjopen-2019-031572

HANDLING MISSING DATA

The percentage and absolute withdrawal of participants lost-to-follow up will be reported for each study arm in the CONSORT flow-chart and reasons for missing data will be documented.

Primary analysis:

Where it is not possible to obtain full follow-up data for the primary outcome, baseline observations will be carried forward. We will assess the sensitivity of the analysis to assumptions about missing data by also running an analysis restricted to participants completing follow-up and an analysis imputing the last home-measured weight for people who did not attend the final meeting and did not self-report their weight at eight-week follow-up. All tests will be done at a 5% two-sided significance level.

Secondary analyses:

If participants in the control condition experience issues with the synchronisation of weight measurements to the BodyTrace server, self-reported written records of weighing data will be accepted as measures of weighing adherence. Where control group participants do not keep a written record of measurements, the proportion of days adherent to weighing before the issues with synchronisation arose will be calculated.
Where participants in the intervention condition do not use the weight-tracking app or the app data is faulty, self-reported written records of weight measurements will be accepted as weight-tracking adherence measures. Where the weight-tracking data is incomplete due to technical reasons (e.g. switching of mobile phones), the proportion of days adherent to weight-tracking will be calculated for the time frame covered by the existing data. Missing data will not be imputed for the moderator variables. That is, cases with missing data on adherence and intervention rating will be excluded from the moderator analyses.

HANDLING OUTLIERS

We do not expect significant outliers based on our definition of population for analysis.

MODEL ASSUMPTIONS

For the primary analysis, the normality of all model residuals will be assessed using histograms, QQ-plots and other diagnostic plots. Where the normality assumption is violated, a sensitivity analysis using semi-parametric generalized estimating equations will be run additionally.

PREVAIL Trial Statistical Analysis Plan, Version 1.0, 21/10/2019

APPENDICES

Appendix 1. Template tables for presentation of results

Baseline Demographic Characteristics

N(%), unless otherwise specified	Control (n=)	Intervention (n=)	Total (n=)
Age, years, mean (SD)			
Gender, % female			
BMI, kg/m^2, mean (SD)			
Ethnicity			
White			
Asian or Asian British			
Black of Black British			
Mixed/Other			
Highest Educational Qualification			
No formal qualifications			
Vocational/work-related qualifications			
GCSE, NVQ level 1			
Apprenticeship			
A' levels, NVQ level 2-3			
Other post-high school qualifications			
University Degree, NVQ level 4+			
Employment Status			
Employed			
Self-employed			
Unemployed			
Looking after home and family			
Student			
Retired			
Long-term sick or disabled			

Frie K, *et al. BMJ Open* 2019; 9:e031572. doi: 10.1136/bmjopen-2019-031572

PREVAIL Trial Statistical Analysis Plan, Version 1.0, 21/10/2019

Other Baseline Characteristics

N(%), unless otherwise specified	Control (n=)	Intervention (n=)	Total (n=)
Weighing Frequency			
Less than once a month			
Once a month			
Every other week			
Once a week			
Liking of weighing			
Dislike it a great deal			
Dislike it somewhat			
Neither like nor dislike it			
Like it somewhat			
Like it a great deal			
Usefulness of weighing to control weight			
Definitely not			
Probably not			
I don't know			
Probably yes			
Definitely yes			

Frie K, et al. BMJ Open 2019; 9:e031572. doi: 10.1136/bmjopen-2019-031572

UNIVERSITY OF OXFORD NUFFIELD DEPARTMENT OF PRIMARY CARE HEALTH SCIENCES

Primary Outcome (Effectiveness)

Linear Mixed Effects Model

		Mean (SD) weight change from baseline			
		Intervention	Control	Adjusted difference (95% CI)	*p*
Intention to Treat Analysis	Baseline Observation Carried Forward (N=)				
	Last Home-measured Weight (N=)				
	Completed Follow-up (N=)				
Per Protocol Analysis (N=)					
Adjusted for: GP practice (random effect), baseline weight (fixed effect)					

The Nuffield Department of Primary Care Health Sciences is part of the NIHR School for Primary Care Research. Head of Department: Professor Richard Hobbs FRCGP FRCP FESC FMedSci

Athena SWAN Silver Award

Secondary Outcomes (Process Evaluation)

Adherence Rates

Intervention Component	Mean % (SD)
Control	
Daily Weighing (n=)	
Intervention	
Daily Weighing (n=)	
Weight-Tracking (n=)	
Daily Action Planning Questionnaires (n=)	
Weekly Reflection/Evaluation Questionnaires (n=)	
Action Diary (optional, n=)	

Adherence Rates Control vs Intervention

**Control vs Intervention**	df	*t*	*p*	95% CI
Daily Weighing (N=)				

NUFFIELD DEPARTMENT OF PRIMARY CARE HEALTH SCIENCES

Intervention Component Rating (only intervention condition)

Question	Mean (SD)
1. How do you feel about weighing yourself overall? (n=)	
2. How useful did you find the intervention for controlling your weight overall? (n=)	
3. How useful did you find tracking your weight for controlling your weight? (n=)	
4. How useful did you find planning weight loss actions for controlling your weight? (n=)	
5. How useful did you find reflecting on the reasons for weight changes for controlling your weight? (n=)	
6. How useful did you find the weekly action evaluation for controlling your weight? (n=)	

Questions were completed by members of the intervention condition who attended the follow-up meeting. Question 1 was rated on a scale from 1 (very negative) to 10 (very positive). Questions 2-6 were rated on a scale from 1 (not useful) to 10 (very useful).

The Nuffield Department of Primary Care Health Sciences is part of the NIHR School for Primary Care Research. Head of Department: Professor Richard Hobbs FRCGP FRCP FESC FMedSci

Athena SWAN Silver Award

UNIVERSITY OF OXFORD
NUFFIELD DEPARTMENT OF PRIMARY CARE HEALTH SCIENCES

Moderators of Effectiveness

Linear Mixed Effects Models

<table>
<tr><td colspan="2" rowspan="2"></td><td colspan="3">Adjusted Difference (95% CI); p</td></tr>
<tr><td>Condition</td><td>Additional Variable</td><td>Additional Variable*Condition</td></tr>
<tr><td rowspan="4">Moderator Variables</td><td>Adherence (N=)</td><td></td><td></td><td></td></tr>
<tr><td>University degree (N=)</td><td></td><td></td><td></td></tr>
<tr><td>Liking of weighing (N=)</td><td></td><td></td><td></td></tr>
<tr><td>Overall intervention rating (N=)</td><td>-</td><td></td><td>-</td></tr>
<tr><td colspan="5">Outcome: Weight Change (BOCF); adjusted for: GP practice (random effect), baseline weight (fixed effect)</td></tr>
</table>
