# Supplementary material for: Testing the effectiveness of a weight loss intervention to enhance self-regulation in adults who are obese: protocol for a randomised controlled trial
Source: BMJ Open. 2019 Dec 8;9(12):e031572. doi: 10.1136/bmjopen-2019-031572 (PMC6924834; doi:10.1136/bmjopen-2019-031572)
Supplement: Author's manuscript [file bmjopen-2019-031572.draft_revisions.pdf]

BMJ Open

BMJ Open is committed to open peer review. As part of this commitment we make the peer review history of every article we publish publicly available.

When an article is published we post the peer reviewers' comments and the authors' responses online. We also post the versions of the paper that were used during peer review. These are the versions that the peer review comments apply to.

The versions of the paper that follow are the versions that were submitted during the peer review process. They are not the versions of record or the final published versions. They should not be cited or distributed as the published version of this manuscript.

BMJ Open is an open access journal and the full, final, typeset and author-corrected version of record of the manuscript is available on our site with no access controls, subscription charges or pay-per-view fees (http://bmjopen.bmj.com).

If you have any questions on BMJ Open's open peer review process please email info.bmjopen@bmj.com

BMJ Open

# Testing the effectiveness of a weight loss intervention to enhance self-regulation in adults who are obese: protocol for a randomised controlled trial



| | |
|---|---|
| Journal: | *BMJ Open* |
| Manuscript ID | bmjopen-2019-031572 |
| Article Type: | Protocol |
| Date Submitted by the Author: | 10-May-2019 |



| | |
|---|---|
| Complete List of Authors: | Frie, Kerstin; University of Oxford, Nuffield Department of Primary Care Health Sciences<br>Hartmann-Boyce, Jamie; University of Oxford, Nuffield Department of Primary Care Health Sciences; Nuffield Department of Primary Care Health Sciences, Oxford University<br>Jebb, Susan; University of Oxford, Nuffield Department of Primary Care Health Sciences<br>Aveyard, Paul; University of Oxford, Nuffield Department of Primary Care Health Sciences |



| | |
|---|---|
| Keywords: | self-regulation, self-monitoring, self-experimentation, weight loss, obesity |
| | |

SCHOLARONE™ Manuscripts

# Testing the effectiveness of a weight loss intervention to enhance self-regulation in adults who are obese: protocol for a randomised controlled trial

Kerstin Frie[1], Jamie Hartmann-Boyce[1], Susan A Jebb[1], Paul Aveyard[1]

[1] Nuffield Department of Primary Care Health Sciences, University of Oxford, UK

Corresponding Author: Kerstin Frie
E: kerstin.frie@phc.ox.ac.uk, T: +44 (0) 1865 289317, Postal address:
Nuffield Department of Primary Care Health Sciences, University of Oxford, Radcliffe Primary Care Building, Radcliffe Observatory Quarter, Woodstock Road, Oxford, OX2 6GG

Word count: 3999

For peer review only

## ABSTRACT

**Introduction:** Previous trials finding an effect of self-monitoring on weight loss have considered the effect to be mediated by self-regulatory processes. However, a qualitative think-aloud study asking people to record thoughts and feelings during weighing showed that self-regulation occurs only rarely without further instruction. The aim of this trial is to test a novel intervention which guides people through the self-regulatory processes to see whether it facilitates weight loss.

**Methods and analyses:** A parallel group, randomised controlled trial will be conducted to test the concept that a self-regulation intervention for weight loss increases weight loss compared to daily self-weighing without further support. One hundred participants with a BMI≥30 kg/m$^2$ will be randomised to either the control or intervention group. The control group will be asked to weigh themselves daily for eight weeks, the intervention group will be encouraged to follow the self-regulation intervention. They will be prompted to weigh daily, track their weight using an app, plan daily actions for weight loss and reflect on their action plans on a weekly basis. This self-regulation cycle will allow them to experiment with different weight loss strategies and identify effective and sustainable actions. Primary and process outcomes will be measured at baseline and 8-weeks follow-up. Linear regression analysis of the primary outcome, weight change, will assess the early effectiveness of the intervention. The process outcomes liking, perceived effectiveness, as well as usage and barriers with regards to the PREVAIL intervention, will be assessed through qualitative analysis of follow-up interviews and quantitative analysis of adherence rates and responses to a final questionnaire.

**Ethics and dissemination:** This trial was reviewed and approved by the NHS National Research Ethics Committee and the Health Research Authority (reference number: 18/SC/0482). The findings of the trial will be published in peer reviewed journals and presented at conferences.

**Trial registration number:** ISRCTN14148239, Pre-recruitment.

**Protocol Version:** Version 1.1, 7th of December 2018

**Keywords:** self-regulation, self-monitoring, self-experimentation, weight loss, obesity



For peer review only - http://bmjopen.bmj.com/site/about/guidelines.xhtml

## STRENGTHS AND LIMITATIONS OF THIS STUDY

**Strengths**

- This trial will provide preliminary evidence for whether a remotely delivered and iterative self-regulation intervention can enhance weight loss
- The intervention addresses specific barriers to self-regulation, identified in a previous qualitative study of experiences of self-weighing
- Process evaluation measures will assess participants liking, usage and perceived effectiveness of the intervention to enable future improvements

**Limitations**

- Blinding might be compromised as participants might be aware that they were assigned to the control or treatment group
- The study is designed as a 'proof-of-concept' trial and longer-term studies will need to evaluate the effectiveness of the intervention as a weight management tool

For peer review only



For peer review only - http://bmjopen.bmj.com/site/about/guidelines.xhtml

## INTRODUCTION

Self-monitoring of weight is often employed in multi-component weight loss interventions, as evidence suggests it aids weight loss(1-5). The weight loss effect has often been ascribed to a self-regulation mechanism, based on the hypothesis that self-monitoring triggers self-regulation(1, 4-6). In this context, self-regulation occurs in iterative cycles, starting by (1) contextualising the weight with previous measurements and goals, thus providing (2) an opportunity to reflect on previous behaviour and reinforce successful actions, enabling (3) the planning of actions to reach the goal, followed by (4) the performance of planned actions(6-8). This cycle of processes allows for experimentation with different weight loss techniques, helping the user to build a personal portfolio of effective and sustainable strategies(9, 10).

A study employing self-weighing as a standalone intervention did not find a significant weight loss effect, raising the question whether self-regulation is performed naturally after weighing or whether additional weight loss treatment components are necessary(11). We addressed this question in a think-aloud study, where twenty-four participants were asked to record their thoughts and feelings during daily weighing for eight weeks, without being prompted to self-regulate(12). On 90% of occasions, participants contextualised their weight measurement and on 58% participants reflected on previous behaviours. Only on 20% of occasions did participants plan actions and specific action-planning, defining a concrete action and time plan, was rare (6%). The frequency of specific action-planning was, however, significantly predictive of weight loss. Hence, the study provided support to the notion that completing the last step of the self-regulation process can elicit weight loss. However, the think-aloud study also showed that self-regulation does not occur autonomously, and that people need support in developing self-regulation skills, especially action-planning. Self-regulation, once learnt, has the potential to be a weight loss strategy that is performed autonomously and sustainably. It provides an opportunity for remote weight loss interventions that do not require resource-intensive support from health care providers.

Several weight loss interventions including self-regulation components have been developed over the years. Some educate their users about self-regulation theory without providing active support for the key component - specific action planning(1, 4, 13, 14). Others support users in action planning, but fail to imitate the iterative nature of self-regulation, as they do not allow the usage of self-monitoring feedback for adaptations to the action plans(15-18), thus disabling self-regulation. Some interventions guide participants through iterative reformulation of action plans following self-monitoring feedback, but are delivered through face-to-face sessions(19, 20), which are resource-intensive and not easily rolled out at large scale. Other interventions incorporating action



For peer review only - http://bmjopen.bmj.com/site/about/guidelines.xhtml

planning dictate which actions participants are supposed to follow(15, 21, 22), which might reduce goal ownership, a significant predictor of goal engagement and attrition(23, 24). Notwithstanding these critiques, seven of the eleven studies cited here found significant weight loss, suggesting that components targeting the self-regulation cycle can enhance weight loss, encouraging further work in this area. Our critiques highlight the need for an intervention that guides participants through the whole and iterative self-regulation process in an autonomous, low-cost and scalable manner. Furthermore, since many interventions add self-regulation elements to a broader spectrum of weight loss treatment components(14, 17, 25-27), a study testing self-regulation as a standalone intervention is needed to investigate whether iterative self-regulation is sufficient to achieve weight loss.

With this study we aim to test the proof of concept of an intervention aiming to address this gap in the literature. The PREVAIL intervention (**P**eople **RE**gulating themsel**V**es to **A**chieve we**I**ght **L**oss) is a weight loss programme guiding people through the iterative self-regulation process. It encourages users to experiment with different weight loss approaches, and use the self-regulation mechanism to find their ideal set of tools.

## OBJECTIVES

The primary objective of this trial is to test the concept whether an intervention, which trains individuals in self-regulatory processes, aids early weight loss in comparison to unsupported daily weighing. Other objectives pertain to the evaluation of usage and effectiveness of the self-regulation intervention components, as well as the qualitative analysis of participant experiences of the intervention.

## METHODS

### Study design and setting

An individually randomised, two arm, parallel group design will be employed, assessing superiority of the self-regulation intervention over daily self-weighing alone. Participation will last eight weeks. Participants will attend two study visits, one at baseline and one after the end of the 8th week of the intervention. The primary outcome will be weight change. The study will take place in Oxfordshire, UK.

### Recruitment

Two to four general practitioner (GP) practices around Oxford, UK, will function as participant identification centres and search their health records to identify



For peer review only - http://bmjopen.bmj.com/site/about/guidelines.xhtml

suitable patients for the trial (age ≥ 18 years, BMI ≥30kg/m$^2$). The GP will screen the search list and exclude patients who would be inappropriate to invite, including terminally ill or violent patients. Suitable patients will be sent an invitation letter from their GP. They will be encouraged to contact the research team if they are interested in taking part. GPs may also identify suitable patients during routine consultations. We will ask practices not to refer participants to commercial weight loss programmes, other obesity clinics or bariatric surgery, whilst they are enrolled as participants in this trial.

**Eligibility criteria**

Inclusion Criteria

- Participant is willing and able to give informed consent
- Aged 18 years or above
- BMI ≥30 kg/m$^2$
- Owns an Apple or Android smartphone

Exclusion Criteria

The participant may not enter the study if any of the following apply:

- Unable to understand English
- Unable to follow all intervention procedures for a period of more than 4 consecutive days
- Currently self-monitoring body weight more than once a week
- Currently or within three months of study entry attended a weight management programme or currently participating in another weight loss study
- Lost more than 5% of current body weight in the last six months
- Prior bariatric surgery, or scheduled for bariatric surgery
- Pregnant, or planning to become pregnant during the course of the study
- Have an electronic medical implant, such as a pacemaker
- Have ever had or been diagnosed with an eating disorder
- People that the GP judges not able to meet the demands of either treatment programme or measurement schedule. This may include severe medical problems not listed above.

**Participant flow**

Screening

People who are interested in taking part will contact the research team. The research team will then discuss study participation by telephone or email and undertake screening. If the person appears eligible and would like to attend a baseline visit, the research team will offer an appointment at a local venue. The participants will be emailed a participant information sheet (PIS).



For peer review only - http://bmjopen.bmj.com/site/about/guidelines.xhtml

Baseline

When the participants attend the baseline appointment, a member of the research team will seek informed consent and check eligibility for inclusion in the study by measuring height, weight and body composition for BMI calculation. The participants will be asked to complete an online questionnaire, capturing demographics (i.e., age, gender, ethnicity) and previous experiences with self-weighing. Participants will be randomised and receive instructions for the assigned intervention. The researcher will provide participants with a body scale for the duration of the trial. A follow-up appointment will be scheduled for after the completion of the intervention period.

Follow up

The aim of the follow-up appointment is to assess the outcomes of the trial. The research team will email participants in advance to remind them of the meeting. In the intervention group, this email will also contain a final questionnaire, asking participants about the usefulness of the intervention components.
The appointment will be scheduled at a local venue and conducted by a member of the research team. Participants will be asked to return the body scales. The researcher will measure weight and body composition.
Twenty participants in the intervention group will be purposively sampled and invited to participate in a semi-structured interview at the end of the follow-up meeting. The sample chosen will aim to reflect different levels of adherence, weight change, and responses to the final questionnaire. Participants will be asked about their experiences with self-weighing, their liking of the intervention components, as well as perceived barriers to engaging with the intervention. Interviews will be recorded, transcribed and analysed. A study flow chart is displayed in Figure 1.

Insert Figure 1 here

Figure 1: Study Flow Chart

**Sample size**

We intend to recruit 100 participants, which is sufficient to detect a 1.5kg difference between conditions, at 90% power and 5% type I error rate, while allowing for a 20% drop-out rate. Variance of weight change was based on results of a similar trial(28), which reported a standard deviation of 2.13kg at 2 months follow-up.



For peer review only - http://bmjopen.bmj.com/site/about/guidelines.xhtml

**Randomisation and blinding**

All eligible participants will be randomised with an allocation ratio of 1:1 to the intervention or control group. A randomisation sequence, stratified by GP and using block randomisation with randomly varying block sizes of 2 and 4 will be generated using a computer algorithm. Allocations will be concealed in numbered, sealed, opaque envelopes by an independent researcher in the department and handed to the researcher who will conduct the baseline visits. Due to the nature of this trial, it will be difficult to blind participants to the treatment allocation. We will aim to make it as opaque as possible by presenting daily weighing as an intervention to control group participants, as done in previous trials(29). The researchers conducting baseline and follow-up will perform data analysis, and will not be blinded to treatment allocation. The primary outcome, weight change, will be measured objectively. Adherence to self-regulation steps will be measured objectively through the frequency of weight logs and completed questionnaires in control and intervention group. The evaluations of treatment components in the final questionnaire will be measured without researchers' input and analysed quantitatively. Blinding of the researcher who conducts and analyses the semi-structured interviews with intervention group participants will not be possible.

**Intervention and control**

Intervention

Participants will be asked to weigh themselves every morning after waking using standard body scales provided to them. In addition, they will be prompted to complete tasks which speak to the different steps of the self-regulation process, including (1) contextualising the weight measurement, (2) reflecting on behaviour, and (3) planning weight loss actions. The individual tasks and their development are described in more detail below. Input from members of the public was sought at several stages of the intervention development.

(1) Contextualising: In the think-aloud study participants struggled memorising daily measurements and keeping an overview of their weight loss progress, which impeded their ability to use weight measurements as constructive feedback. Participants stated they would have benefitted from a weight-tracking tool. Therefore, to support participants in contextualising their weight measurements, we will encourage them to use the app "Weight Loss Tracker, BMI" by aktiWir GmbH. Research shows that digital tracking devices can significantly increase adherence to self-monitoring(30, 31), perhaps because the visualization of progress and feedback on weight loss success provides motivation and keeps users on track with their goals(32).

(2) Reflecting: The think-aloud study showed that participants struggled to interpret day-to-day weight changes due to daily fluctuations that were not caused by fat loss or gain(12). We therefore 111decided to encourage reflection



For peer review only - http://bmjopen.bmj.com/site/about/guidelines.xhtml

on a weekly rather than daily basis. Participants will receive weekly emails with feedback on their weekly weight trends, asking them to complete an online questionnaire (Qualtrics, USA). The questionnaire will prompt reflection on the relationship between behaviours performed throughout the week and weight change observed. Participants will be asked to use this insight to evaluate the use of the weight loss actions they had performed throughout the week.

(3) Planning: We aimed to strike a balance between ensuring that participants choose appropriate actions and allowing them to choose actions themselves, as lack of goal ownership predicts attrition(23). Participants will therefore choose one weight loss action per day from a list of 53 actions (see Appendix 1). To create the actions list, we first identified weight loss actions from effective weight loss interventions in the literature. These were reviewed, adapted and complemented during iterative brainstorming sessions with an interdisciplinary expert team, comprised of dietitians, general practitioners and psychologists.

The rationale for daily action plans was twofold: 1) allowing participants to adapt their actions flexibly to their day and 2) giving participants exposure to a wider range of strategies.

Based on action planning and implementation intention research, which shows that the specificity of action plans increases likelihood of implementation(33-35), we will ask participants to specify where, when and how they will perform their chosen action, which cues they will use, which barriers they might experience and how they will deal with them.

As participants only reflect on their behaviour on a weekly basis, we grouped the actions conceptually into seven categories, five of which cover diet-related actions and two of which cover physical activity-related actions. Participants will be asked to choose one category at the beginning of the week, and choose daily actions from this category for the rest of the week. Weekly behaviour reflection will therefore focus on the effectiveness of a category of actions. Taken together, the reflection and action planning process shall enable participants to experiment with different weight loss approaches and decide on their effectiveness and usefulness based on trends in weight data.

At the beginning of weeks two through eight of the intervention, participants will be prompted to commit to some of the actions of previous weeks, as the performance of several weight loss actions increases chances for weight loss.

Action planning support will be provided through daily questionnaires (Qualtrics, USA), which will be sent to participants every morning via email.

Participants will receive detailed instructions and a manual for the intervention at baseline. They will also be given an action diary, in which they will be asked to record their performed weight loss actions.

We will call participants at the end of the 1st and 4th week to ask about and solve any technical problems that may have arisen (e.g. with the functioning of the



For peer review only - http://bmjopen.bmj.com/site/about/guidelines.xhtml

scales or the receiving of questionnaires). A figure from the study manual depicting the intervention procedure is displayed in Figure 2. The TiDIER checklist(36) for the PREVAIL intervention and the comparator is reported in Table 1.

Insert Figure 2 here

Figure 2: Intervention procedure as depicted in study manual

Table 1: TiDIER checklist describing the intervention and control condition

| | Intervention: PREVAIL | Control: Daily Self-Weighing without behavioural support |
|---|---|---|
| BRIEF NAME | PREVAIL (**P**eople **Re**gulating Themsel**v**es to **A**chieve We**i**ght **L**oss) | Self-Weighing Only |
| WHY | **Self-weighing:** Monitoring weight on a daily basis will enable participants to take note of their weight loss progress.<br>**Weight-Tracking:** Our preceding study(12) found that people can lose track of their weight loss progress when weighing every day because they struggle to remember measurements. We therefore ask participants to track their weight.<br>**Action Planning:** In our preceding study, participants rarely made action plans to help them progress with their weight loss(12). If they did make action plans, they were rarely specific. Specificity of action plans is a significant predictor of the likelihood of implementation(33-35). We therefore guide participants through a specific action planning process.<br>**Report Email:** Participants in our preceding study struggled to see trends in their weight data(12). Unfortunately, the app we are using for weight-tracking does not provide users with trend information. We will therefore send out weekly emails, containing a statement about the trend of the weight measurements of the last week.<br>**Reflection and Action Evaluation:** Our preceding study revealed that daily fluctuations vary over time within people, making it difficult to interpret daily weight changes. We therefore want to encourage participants to reflect on their weight changes and the effectiveness of their weight loss actions on a weekly basis. Using the weekly weight trend information, participants will be able to evaluate whether they found the group of actions they performed effective and worth repeating. | A previous trial has found that self-monitoring of weight without further guidance is not effective for weight loss.(11) |
| WHAT | **Self-weighing:** Participants will be instructed to weigh themselves daily using provided body scales. They will be asked to weigh themselves in a similar state every day, ideally first thing in the morning and without clothes.<br>**Weight-Tracking:** Participants will be asked to download the free app "Weight Loss Tracker, BMI" by aktiWir GmbH on their smartphone and use it on a daily basis to record their weight measurements. They will be asked to submit a backup of their data to the research team every week.<br>**Action Planning:** Participants will receive a daily questionnaire helping them to plan a weight loss action. At the beginning of first week they will be asked to choose a | Self-weigh every morning, in a similar state. |

For peer review only



For peer review only - http://bmjopen.bmj.com/site/about/guidelines.xhtml

| | | |
|---|---|---|
| | category of actions, and then they will be able to choose one of the actions within this category per day for the rest of the week. There are seven categories in total, five covering diet related actions and two covering physical activity related actions. Participants will be asked to specify for each action how, when, and where they are going to perform it, and which cues they are going to use. They will also be prompted to think about how to overcome potential barriers. At the start of weeks 2 through 8, participants will be encouraged to try out a new category of actions. We will additionally ask participants on a weekly basis to commit to continuing some of the actions they tried out in previous weeks. In order to help participants to maintain an overview of the actions they performed so far, we will provide them with a non-digital action plan diary.<br>**Report Email:** Once a week, participants will receive an email from the research team, informing them about their trend weight change for the last week. This trend weight change consists of the slope of a trend line fitted across all measurements of the week, multiplied by the number of days covered by the measurements.<br>**Reflection and Action Evaluation:** In the weekly report email, participants will receive a link to the reflection and action evaluation questionnaire. This questionnaire prompts participants to think about why their weight has changed as it has. They are further asked to evaluate the group of actions they performed across the week, including whether they found them useful and whether they would repeat them. On the basis of this evaluation, participants will be able to decide which actions they want to continue doing in the next weeks, and which ones to drop. Using this method of self-experimentation, participants will be able to try and test different weight loss strategies and identify the ones that are effective and sustainable for them. | |
| WHO | The chief investigator (KF) will deliver the intervention at the baseline session. She will also organise the mailing of all questionnaires and weekly report emails. She will be the primary contact for all participants. KF is a psychologist by background and has received GCP training. | The chief investigator (KF) will instruct participants in the control group to weigh themselves every day. |
| HOW | Participants will measure their weight on provided body scales. They will use a free weight-tracking app called “Weight Loss Tracker, BMI” by aktiWir GmbH on their smartphone to track their weight loss progress. All questionnaires will be sent automatically to participants in the early morning by the survey platform Qualtrics (USA). | Participants measure their weight on provided body scales. |
| WHERE | At home. | At home. |
| TAILORED | Participants are able to tailor the intervention to themselves by choosing action plans relevant to them. | N/A |
| HOW Well | We will request participants to send a back-up of their data in the weight-tracking app once a week. This data will allow us to assess adherence to the weighing and weight-tracking components of the intervention. Completion of the daily action planning and weekly reflection questionnaires will allow us to assess adherence to action planning and refection. | The provided body scales contain a SIM card which automatically transfers the weight data to a secure research server. We will therefore be able to assess adherence. |



For peer review only - http://bmjopen.bmj.com/site/about/guidelines.xhtml

Control

Participants in the control group will be instructed to self-weigh daily. By using this comparator group, we want to test whether the self-regulation process can enhance self-weighing to be an effective weight loss tool. Participants will receive smart scales (BodyTrace, Inc., New York) which are equipped with a SIM card and automatically transfer measurements to a secure server via the 3G/4G network. This will allow us to assess adherence to daily weighing in the control group.

**Patients and public involvement**

Members of the public were involved in the design of the study at several stages. After creating the invitation letter, PIS, informed consent form, semi-structured interview guide, as well as the baseline, daily, weekly and follow-up questionnaires for the PREVAIL intervention, we asked members of the public for feedback. Our department has a panel of >100 members of the public with an interest in weight management. Based on phone calls with members of this panel we were able to make the materials clearer and more concise. The manual, explaining the intervention in detail, was further discussed with members of the panel in a focus group session. As a result of this focus group session, the manual was professionally edited to be shorter. We also added figures and graphs to present the procedures of the study more visually. The panel helped to revise the explanations of the different action plans.

A test run of four weeks was conducted with five members of the department, who are not otherwise involved in this study. They provided feedback on the running of the intervention, which, amongst other outcomes, led to the creation of a reminder email which will be sent out before the start of the intervention.

No members of the public will be involved in conducting or analysing the study. However, we will gather input on the most suitable and effective ways to disseminate our research findings to the public.

**Outcomes**

Primary outcome

- Change in body weight between baseline and follow-up by condition

Process Evaluation

- Adherence to self-regulation steps, assessed through weight records and daily action-planning/weekly evaluation questionnaires
- Action plan use and evaluation, based on information from daily and weekly questionnaires, and follow-up interviews
- Perceived effect of intervention and liking of intervention features based on final questionnaire and follow-up interviews
- Barriers and unmet needs for successful weight loss, assessed in follow-up interviews



For peer review only - http://bmjopen.bmj.com/site/about/guidelines.xhtml

## Measurements

A schedule of measurements can be found in Table 2.

Physical measurements

Participants' height will be measured at baseline to the nearest 0.1cm using a stadiometer. Weight and body composition will be measured both at baseline and follow-up using a body composition scale (SC-240 MA, Tanita Japan), which measures body composition using bioelectrical impedance. Weight will be recorded to the nearest 0.1kg and body fat to the nearest 0.1%.

Process Evaluation Measures

*Adherence measures*

For the control group, adherence to daily weighing will be measured by calculating the proportion of days for which we have a recorded weight measurement on the server.

For the intervention group, adherence to daily weighing and weight-tracking will be assessed by calculating the proportion of days for which a weight was recorded in the weight-tracking app or in the daily action-planning questionnaire. Adherence to action planning and reflection will be measured by calculating the proportion of days on which the respective questionnaires were completed.

*Action Plan Use and Evaluation*

For each action plan and action category, we will calculate how often each participant used it across the eight weeks. We will score how often each category of actions was evaluated as useful, partly useful or not useful in the reflection and evaluation questionnaire.

*Evaluation of intervention components*

Using the data from the final online questionnaire, we will calculate average ratings of the intervention components.

For peer review only

Table 2: Schedule of measurements.

| | Screening | Baseline visit | Intervention Period | Completion email | Follow-up visit (after 8 weeks) |
|---|---|---|---|---|---|
| Length | 10 mins | Up to 1h | 10 mins per day | 5 mins | up to 45 mins |
| Who conducts | Research Team | Research Team | Participants | Research Team | Research Team |
| Eligibility assessment | ☑ | ☑ (BMI) | | | |
| Enrolment | | ☑ | | | |
| Baseline questionnaire | | ☑ | | | |
| Weight | | ☑ | ☑ | | ☑ |
| Height | | ☑ | | | |
| Body composition | | ☑ | | | ☑ |
| Allocation | | ☑ | | | |
| Weight-tracking (only intervention) | | | ☑ | | |
| Daily Questionnaire (only intervention) | | | ☑ | | |
| Weekly report and questionnaire (only intervention) | | | ☑ | | |
| Final questionnaire (only intervention) | | | | ☑ | |
| Semi-structured interview (20 participants in intervention group) | | | | | ☑ |

**Retention**

The daily questionnaire and weekly report emails will act as prompts for the participants to engage with the intervention. At the end of the follow-up visit, participants will receive a £35 one4all gift card.

There are no criteria for withdrawal other than participants' request to withdraw. Participants can also ask to withdraw their collected data. We will ask participants wishing to withdraw whether they are willing to attend the final follow-up and take part in an exit interview to understand the reasons for their discontinuation.

**Statistical analyses**

The statistical analysis of the primary outcome, effectiveness of the intervention for weight loss, will be carried out on the basis of intention-to-treat (ITT). That is, after randomisation, participants will be analysed according to their allocated intervention group. We will endeavour to obtain full follow-up data on every participant to allow full ITT analysis. A linear regression, predicting weight at 8-weeks follow-up while adjusting for baseline weight and GP practice, will assess



For peer review only - http://bmjopen.bmj.com/site/about/guidelines.xhtml

the effect of condition(37). We will assess the sensitivity of the analysis to different assumptions about missing data using a variety of imputation methods, including baseline observation carried forward analysis and an analysis of participants completing follow-up. A final analysis will impute the last home-measured weight for people who did not attend the final follow-up. All tests will be done at a 5% two-sided significance level.

The process evaluation measures of the intervention and participant experiences will be analysed in several ways. Adherence rates will be assessed and compared between the experimental and control condition. Actions chosen in the action planning task will be assessed in context with their evaluation in the weekly reflection questionnaire and the resulting weight loss success to identify more and less used, as well as more and less effective strategies.

Further exploratory analyses may be added post-hoc based on preliminary findings. A more detailed analysis plan will be written before analysis commences.

**Qualitative study**

All interview audio-recordings will be transcribed and entered into the NVivo software package (QSR International) for qualitative data analysis. Framework analysis according to Ritchie and Spencer(38) will assess the participants' experiences and perceptions of the different intervention components. The findings will be put into context with the results from the final questionnaire. Inductive thematic analysis following Braun and Clarke(39) will explore additional themes, including barriers and unmet needs. Coding for the framework and thematic analysis will be performed by two independent researchers for a subset of the data to establish reliability. If acceptable reliability is reached ($\kappa > .70$), one researcher will perform the remaining coding.

**Trial management group**

The day-to-day management and operation of the study will be coordinated by KF. A Trial Management Group (TMG), consisting of the authors of this paper, will have oversight of the trial. The TMG will be responsible for the monitoring of all aspects of the trial's conduct and progress and will ensure that the protocol is adhered to and that appropriate action is taken to safeguard participants and the quality of the trial itself. The TMG will meet regularly throughout the course of the trial.

**Adverse events**

This is a low risk trial where it is implausible that the intervention will lead to differences in the occurrence of adverse events so we decided that it was inappropriate to burden participants to collect and record these.



For peer review only - http://bmjopen.bmj.com/site/about/guidelines.xhtml

## Trial monitoring

This is a short trial with no adverse event monitoring or stopping rules so we deemed that a trial steering committee and a data monitoring committee were unnecessary.

## Data management

Data will be kept in accordance with GCP, the Data Protection Act 2018 (DPA) and General Data Protection Regulation (GDPR). Two separate databases will be created, one containing all participant identifiable information, the other capturing all outcome data in an anonymised manner, using a unique participant ID. Weight, height and body composition measurements will be entered into the second database by the researcher. Data from the online questionnaires will be downloaded from Qualtrics and added to the second database. The two databases as well as the anonymised recordings and transcriptions from follow-up interviews will be stored on the secure departmental drive and will only be accessible by members of the TMG. After a lay summary of results has been sent out to participants, the database with participant identifiable data will be destroyed. We will retain the anonymised database for future secondary analyses.

Direct access to study data will be granted to authorised representatives from the sponsor for monitoring and/or audit of the study to ensure compliance with regulations. This access, the reason for it and who has authorised it will be recorded by the TMG. Otherwise, confidentiality will be maintained and no-one outside the TMG will have access to the database.

## Ethics and dissemination

This trial was reviewed and approved by the NHS National Research Ethics Committee and the Health Research Authority (reference number: 18/SC/0482). Any substantial changes to the protocol will be submitted as an amendment to these institutions, as well as the Sponsor. Upon completion of the trial, KF will submit an End of Study notification and final report to the REC Committee, HRA, and Sponsor.

We intend to publish the results of this study in peer reviewed journals, regardless of the nature of the outcome. Authorship will be determined in accordance with the ICMJE guidelines. We will also present our findings at national and international conferences, and publicise our publications through the departments' online presence. Participants will be informed of the trial results through an information sheet prepared for a lay audience. We will also inform our PPI panel members about the findings of the study through their regular newsletter.



For peer review only - http://bmjopen.bmj.com/site/about/guidelines.xhtml

**Acknowledgements**

We would like to acknowledge and thank the members of the public who have helped design the PREVAIL intervention. We thank members of the department who have contributed in expert brainstorming sessions to the development of the intervention. We would like to thank Carmen Piernas for creating the randomisation sequence and the randomisation envelopes. We thank the Clinical Trials Unit of the Nuffield Department of Primary Care Health Sciences for their help with setting up this study and support in conducting the trial.

**Author Contributions**

KF, JHB, SJ, and PA contributed to the design of the intervention and this study. KF led the preparation of the trial. All authors commented and worked on this paper.

**Funding**

This research is funded by the National Institute for Health Research (NIHR) Collaboration for Leadership in Applied Health Research and Care Oxford (CLAHRC) at Oxford Health NHS Foundation Trust. KF's time on this project is funded by NIHR CLAHRC Oxford at Oxford Health NHS Foundation Trust, Wolfson College, University of Oxford (Oxford-Wolfson Marriott-Primary Care Graduate Scholarship), and NIHR School for Primary Care Research (NIHR SPCR). JHB's, SJ's and PA's time on this project is funded by the NIHR Oxford Biomedical Research Centre (BRC) and Oxford CLAHRC. PA and SJ are NIHR senior investigators. The views expressed are those of the authors and not necessarily those of the NHS, the NIHR, the Wellcome Trust, or the Department of Health and Social Care.

**Sponsor**

University of Oxford
University Offices
Wellington Square
Oxford
OX1 2JD
United Kingdom

The sponsor has reviewed all participant-facing documents as part of the ethics application. The sponsor is not involved in the collection, management, analysis, and interpretation of data; writing of the report; and the decision to submit the report for publication of the study.

**Competing interests statement**

The authors have no known competing interests to declare.



For peer review only - http://bmjopen.bmj.com/site/about/guidelines.xhtml

**Licence Statement**

I, the Submitting Author has the right to grant and does grant on behalf of all authors of the Work (as defined in the below author licence), an exclusive licence and/or a non-exclusive licence for contributions from authors who are: i) UK Crown employees; ii) where BMJ has agreed a CC-BY licence shall apply, and/or iii) in accordance with the terms applicable for US Federal Government officers or employees acting as part of their official duties; on a worldwide, perpetual, irrevocable, royalty-free basis to BMJ Publishing Group Ltd ("BMJ") its licensees and where the relevant Journal is co-owned by BMJ to the co-owners of the Journal, to publish the Work in BMJ Open and any other BMJ products and to exploit all rights, as set out in our licence.

The Submitting Author accepts and understands that any supply made under these terms is made by BMJ to the Submitting Author unless you are acting as an employee on behalf of your employer or a postgraduate student of an affiliated institution which is paying any applicable article publishing charge ("APC") for Open Access articles. Where the Submitting Author wishes to make the Work available on an Open Access basis (and intends to pay the relevant APC), the terms of reuse of such Open Access shall be governed by a Creative Commons licence – details of these licences and which Creative Commons licence will apply to this Work are set out in our licence referred to above.

For peer review only

**References**


1. LaRose JG, Gorin AA, Wing RR. Behavioral self-regulation for weight loss in young adults: a randomized controlled trial. Int J Behav Nutr Phy. 2009;6:10.
2. Oshima Y, Matsuoka Y, Sakane N. Effect of weight-loss program using self-weighing twice a day and feedback in overweight and obese subject: a randomized controlled trial. Obesity Research & Clinical Practice. 2013;7(5):e361-6.
3. Helander EE, Vuorinen AL, Wansink B, Korhonen IK. Are breaks in daily self-weighing associated with weight gain? PLoS One. 2014;9(11):e113164.
4. Steinberg DM, Tate DF, Bennett GG, Ennett S, Samuel-Hodge C, Ward DS. The efficacy of a daily self-weighing weight loss intervention using smart scales and e-mail. Obesity (Silver Spring). 2013;21(9):1789-97.
5. Burke LE, Wang J, Sevick MA. Self-monitoring in weight loss: a systematic review of the literature. Journal of The American Dietetic Association. 2011;111(1):92-102.
6. Kanfer FH. Self-Monitoring - Methodological Limitations and Clinical Applications. Journal of consulting and clinical psychology. 1970;35(2):148-52.
7. Boutelle K. Letters to the Editor. Journal of Nutrition Education and Behavior. 2006;38(3):131.
8. Kanfer FH, Karoly P. Self-Control: A Behavioristic Excursion into the Lion's Den. Behav Ther. 1972;3:398-416.
9. Hartmann-Boyce J, Boylan A, Jebb SA, Aveyard P. Experiences of self-monitoring in self-directed weight loss and weight loss maintenance: systematic review of qualitative studies. Qualitative Health Research. 2018:1049732318784815.
10. Daskalova N, Desingh K, Papoutsaki A, Schulze D, Sha H, Huang J. Lessons Learned from Two Cohorts of Personal Informatics Self-Experiments. Proceedings of the ACM on Interactive, Mobile, Wearable and Ubiquitous Technologies. 2017;1(3):46.
11. Madigan CD, Jolly K, Lewis AL, Aveyard P, Daley AJ. A randomised controlled trial of the effectiveness of self-weighing as a weight loss intervention. Int J Behav Nutr Phy. 2014;11:125.
12. Frie K, Hartmann-Boyce J, Jebb S, Pilbeam C, Aveyard P, editors. The role of self-regulation in and barriers to self-monitoring of weight. EHPS 2018; 2018 30.08.2018; Galway, Ireland: EHPS.
13. Pacanowski CR, Levitsky DA. Frequent Self-Weighing and Visual Feedback for Weight Loss in Overweight Adults. Journal of Obesity. 2015;2015:763680.
14. Leahey TM, Thomas G, Fava JL, Subak LL, Schembri M, Krupel K, et al. Adding evidence-based behavioral weight loss strategies to a statewide wellness campaign: a randomized clinical trial. American journal of public health. 2014;104(7):1300-6.


For peer review only



For peer review only - http://bmjopen.bmj.com/site/about/guidelines.xhtml

15. Bennett GG, Steinberg D, Askew S, Levine E, Foley P, Batch BC, et al. Effectiveness of an App and Provider Counseling for Obesity Treatment in Primary Care. American journal of preventive medicine. 2018;55(6):777-86.

16. Dombrowski SU, Endevelt R, Steinberg DM, Benyamini Y. Do more specific plans help you lose weight? Examining the relationship between plan specificity, weight loss goals, and plan content in the context of a weight management programme. Br J Health Psychol. 2016;21(4):989-1005.

17. Benyamini Y, Geron R, Steinberg DM, Medini N, Valinsky L, Endevelt R. A structured intentions and action-planning intervention improves weight loss outcomes in a group weight loss program. American Journal of Health Promotion. 2013;28(2):119-27.

18. Lin M, Mahmooth Z, Dedhia N, Frutchey R, Mercado CE, Epstein DH, et al. Tailored, interactive text messages for enhancing weight loss among African American adults: the TRIMM randomized controlled trial. Am J Med. 2015;128(8):896-904.

19. Huisman S, de Gucht V, Maes S, Schroevers M, Chatrou M, Haak H. Self-regulation and weight reduction in patients with type 2 diabetes: a pilot intervention study. Patient Educ Couns. 2009;75(1):84-90.

20. Mann DM, Palmisano J, Lin JJ. A pilot randomized trial of technology-assisted goal setting to improve physical activity among primary care patients with prediabetes. Prev Med Rep. 2016;4:107-12.

21. Steinberg DM, Levine EL, Askew S, Foley P, Bennett GG. Daily text messaging for weight control among racial and ethnic minority women: randomized controlled pilot study. J Med Internet Res. 2013;15(11):e244.

22. Lin PH, Wang Y, Levine E, Askew S, Lin S, Chang C, et al. A text messaging-assisted randomized lifestyle weight loss clinical trial among overweight adults in Beijing. Obesity (Silver Spring). 2014;22(5):E29-37.

23. Huisman S, Maes S, De Gucht VJ, Chatrou M, Haak HR. Low goal ownership predicts drop-out from a weight intervention study in overweight patients with type 2 diabetes. Int J Behav Med. 2010;17(3):176-81.

24. Deci EL, Ryan RM. The "What" and "Why" of Goal Pursuits: Human Needs and the Self-Determination of Behavior. Psychological Inquiry. 2000;11(4):227-68.

25. Luszczynska A, Sobczyk A, Abraham C. Planning to lose weight: randomized controlled trial of an implementation intention prompt to enhance weight reduction among overweight and obese women. Health Psychology. 2007;26(4):507-12.

26. Linde JA, Jeffery RW, Crow SJ, Brelje KL, Pacanowski CR, Gavin KL, et al. The Tracking Study: description of a randomized controlled trial of variations on weight tracking frequency in a behavioral weight loss program. Contemp Clin Trials. 2015;40:199-211.



For peer review only - http://bmjopen.bmj.com/site/about/guidelines.xhtml

27. Morrison LG, Hargood C, Lin SX, Dennison L, Joseph J, Hughes S, et al. Understanding usage of a hybrid website and smartphone app for weight management: a mixed-methods study. J Med Internet Res. 2014;16(10):e201.
28. Lally P, Chipperfield A, Wardle J. Healthy habits: efficacy of simple advice on weight control based on a habit-formation model. International Journal of Obesity. 2008;32(4):700-7.
29. Madigan CD, Daley AJ, Lewis AL, Aveyard P, Jolly K. Is self-weighing an effective tool for weight loss: a systematic literature review and meta-analysis. Int J Behav Nutr Phy. 2015;12:104.
30. Burke LE, Conroy MB, Sereika SM, Elci OU, Styn MA, Acharya SD, et al. The effect of electronic self-monitoring on weight loss and dietary intake: a randomized behavioral weight loss trial. Obesity (Silver Spring). 2011;19(2):338-44.
31. Carter MC, Burley VJ, Nykjaer C, Cade JE. Adherence to a smartphone application for weight loss compared to website and paper diary: pilot randomized controlled trial. Journal of Medical Internet Research. 2013;15(4):e32.
32. Frie K, Hartmann-Boyce J, Jebb S, Albury C, Nourse R, Aveyard P. Insights From Google Play Store User Reviews for the Development of Weight Loss Apps: Mixed-Method Analysis. JMIR Mhealth Uhealth. 2017;5(12):e203.
33. de Vet E, Oenema A, Brug J. More or better: Do the number and specificity of implementation intentions matter in increasing physical activity? Psychology of Sport and Exercise. 2011;12(4):471-7.
34. Gollwitzer PM, Sheeran P. Implementation Intentions and Goal Achievement: A Meta-analysis of Effects and Processes. Advances in Experimental Social Psychology Volume 38. Advances in Experimental Social Psychology. 382006. p. 69-119.
35. Hagger MS, Luszczynska A. Implementation intention and action planning interventions in health contexts: state of the research and proposals for the way forward. Applied Psychology: Health and Well-Being. 2014;6(1):1-47.
36. Hoffmann TC, Glasziou PP, Boutron I, Milne R, Perera R, Moher D, et al. Better reporting of interventions: template for intervention description and replication (TIDieR) checklist and guide. BMJ. 2014;348:g1687.
37. Vickers AJ, Altman DG. Statistics notes - Analysing controlled trials with baseline and follow up measurements. British Medical Journal. 2001;323(7321):1123-4.
38. Ritchie J, Spencer L. Qualitative Data Analysis for Applied Policy Research. In: Huberman A, Miles M, editors. The Qualitative Researcher's Companion. Thousand Oaks: SAGE Publications, Inc.; 2002. p. 305-29.
39. Braun V, Clarke V. Thematic Analysis. In: Cooper H, Camic, P. M., Long, D. L., Panter, A. T., Rindskopf, D. & Sher, K. J. , editor. APA handbook of research methods in psychology, Vol 2 Research designs: Quantitative, qualitative,



For peer review only - http://bmjopen.bmj.com/site/about/guidelines.xhtml

neuropsychological, and biological. Washington, DC, US: American Psychological Association; 2012. p. 57-71.

For peer review only



For peer review only - http://bmjopen.bmj.com/site/about/guidelines.xhtml

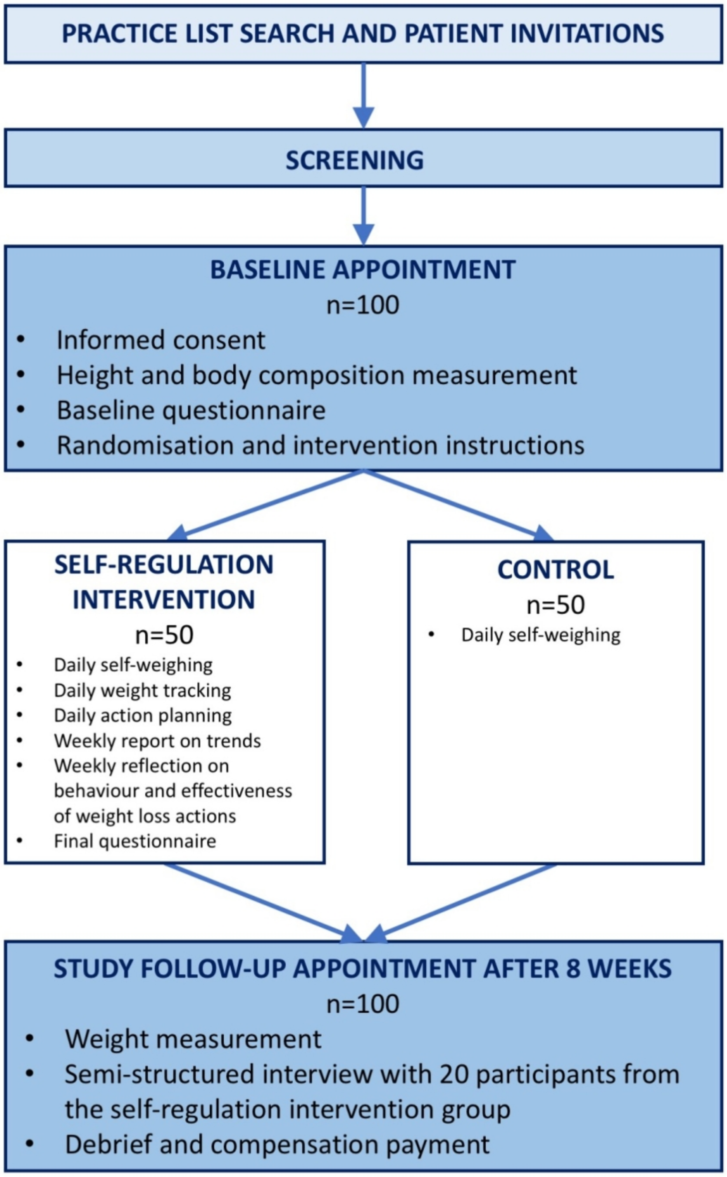


Study Flow Chart

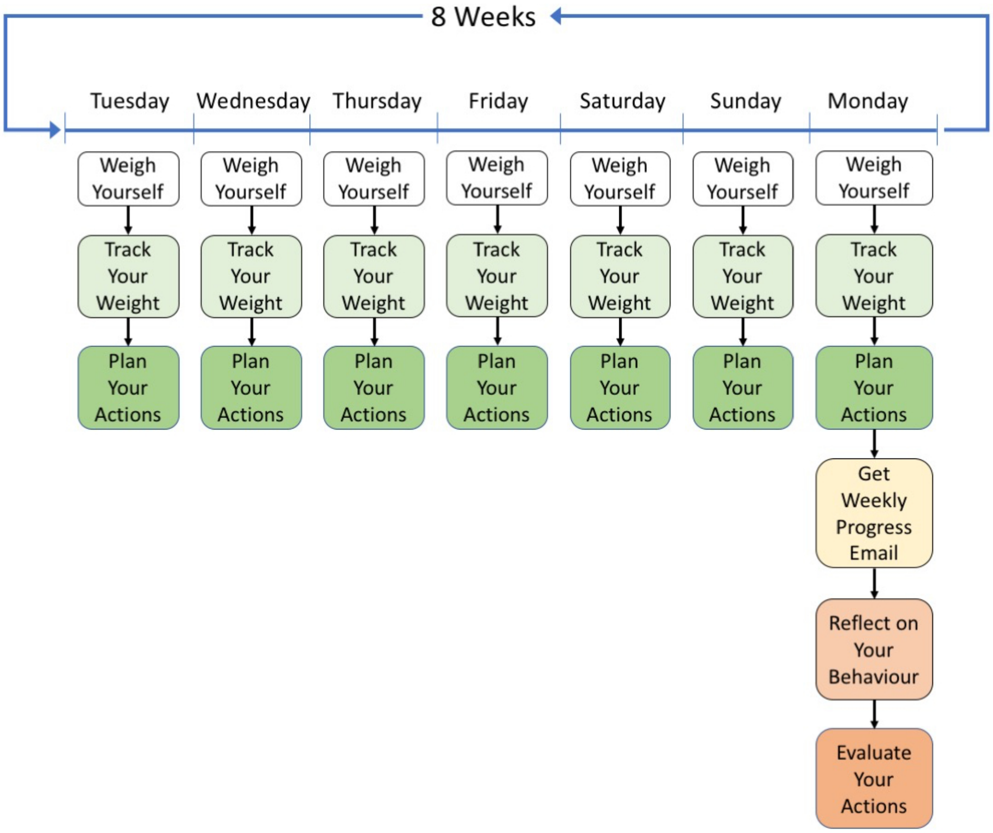


Intervention procedure as depicted in study manual

## List of 53 Weight Loss Actions

Category Red: Eating in a structured way

1. Plan all meals for the day in advance (what and when)
2. Eat no more than three times
3. Skip a meal
4. No calories after 8pm
5. Check the calorie count of everything you want to eat or drink
6. Set yourself a calorie goal and stick to it
7. Have a "fasting" day with less than 800kcal
8. Keep a diary of what you eat and how you feel
9. Check your portion size
10. Only eat when sitting at a table

Category Orange: Avoiding or swapping specific foods

1. Don't eat between meals
2. Cut out crisps, biscuits, cakes and sweets
3. Cut out fried food
4. Have only one course at meal-times
5. Cut out carbs
6. Swap unhealthy snacks for fruits and vegetables
7. Swap rice/potatoes/pasta for extra vegetables
8. Use meal replacement products
9. Swap unhealthy snacks with 6-8 individual nuts

Category Yellow: Changing what you drink

1. Drink only water or unsweetened coffee or tea
2. Swap sugary soft drinks with diet or no sugar versions
3. Do not drink alcohol
4. Drink a pint of water before each meal
5. Swap juices or smoothies with whole fruit and vegetables

Category Green: Creating a healthier diet

1. Eat at least 5 portions of fruit or vegetables each day
2. Snack only on vegetables
3. Eat only foods with a green nutrition label for total fat
4. Eat only foods with a green nutrition label for sugar
5. Make sure half of your main meal of the day is a salad or vegetables
6. Swap rice/potatoes/pasta with extra vegetables
7. Swap fatty meats with lean meats



For peer review only - http://bmjopen.bmj.com/site/about/guidelines.xhtml

Category Blue: Meal-time tactics
1. Eat slowly or 20 chews per bite
2. Focus on your food while eating
3. Stop eating before you feel full
4. Use smaller plates and bowls
5. Cut food into smaller pieces
6. Eat for less than 20 minutes at a time

Category Purple: Burn more calories
1. Walk up and down a flight of stairs for as long as you can
2. Go cycling for as long as you can
3. Go swimming for as long as you can
4. Stretching Exercises
5. Attend an exercise class
6. Play a group sport
7. Go to the gym
8. Exercise at home with the 21-minutes NHS Choices workout
9. Brisk walking for as long as you can

Category Pink: Be more active as part of your daily life
1. Walk 10,000 steps
2. Walk/cycle instead of taking the bus or car
3. Go for a walk with your friend(s)
4. Stand up while working
5. Take the stairs whenever you can
6. Have an active day with your family or friends
7. Stand up while watching TV



For peer review only - http://bmjopen.bmj.com/site/about/guidelines.xhtml

SPIRIT

STANDARD PROTOCOL ITEMS: RECOMMENDATIONS FOR INTERVENTIONAL TRIALS

SPIRIT 2013 Checklist: Recommended items to address in a clinical trial protocol and related documents*

| Section/item | Item No | Description | |
|---|---|---|---|
| **Administrative information** | | | |
| Title | 1 | Descriptive title identifying the study design, population, interventions, and, if applicable, trial acronym | ☑ p. 1 |
| Trial registration | 2a | Trial identifier and registry name. If not yet registered, name of intended registry | ☑ p. 2 |
| | 2b | All items from the World Health Organization Trial Registration Data Set | ☑ p. 1-17 |
| Protocol version | 3 | Date and version identifier | ☑ p. 2 |
| Funding | 4 | Sources and types of financial, material, and other support | ☑ p. 17 |
| Roles and responsibilities | 5a | Names, affiliations, and roles of protocol contributors | ☑ p. 1, 17 |
| | 5b | Name and contact information for the trial sponsor | ☑ p. 17 |
| | 5c | Role of study sponsor and funders, if any, in study design; collection, management, analysis, and interpretation of data; writing of the report; and the decision to submit the report for publication, including whether they will have ultimate authority over any of these activities | ☑ p. 17 |
| | 5d | Composition, roles, and responsibilities of the coordinating centre, steering committee, endpoint adjudication committee, data management team, and other individuals or groups overseeing the trial, if applicable (see Item 21a for data monitoring committee) | ☑ p. 15-16 |
| **Introduction** | | | |
| Background and rationale | 6a | Description of research question and justification for undertaking the trial, including summary of relevant studies (published and unpublished) examining benefits and harms for each intervention | ☑ p. 4-5 |

For peer review only



For peer review only - http://bmjopen.bmj.com/site/about/guidelines.xhtml

| | | | |
|---|---|---|---|
| | 6b | Explanation for choice of comparators | ☑ p. 8-12 |
| Objectives | 7 | Specific objectives or hypotheses | ☑ p. 5 |
| Trial design | 8 | Description of trial design including type of trial (eg, parallel group, crossover, factorial, single group), allocation ratio, and framework (eg, superiority, equivalence, noninferiority, exploratory) | ☑ p. 5 |

**Methods: Participants, interventions, and outcomes**

| | | | |
|---|---|---|---|
| Study setting | 9 | Description of study settings (eg, community clinic, academic hospital) and list of countries where data will be collected. Reference to where list of study sites can be obtained | ☑ p. 5 |
| Eligibility criteria | 10 | Inclusion and exclusion criteria for participants. If applicable, eligibility criteria for study centres and individuals who will perform the interventions (eg, surgeons, psychotherapists) | ☑ p. 6 |
| Interventions | 11a | Interventions for each group with sufficient detail to allow replication, including how and when they will be administered | ☑ p. 8-12 |
| | 11b | Criteria for discontinuing or modifying allocated interventions for a given trial participant (eg, drug dose change in response to harms, participant request, or improving/worsening disease) | ☑ p. 15 |
| | 11c | Strategies to improve adherence to intervention protocols, and any procedures for monitoring adherence (eg, drug tablet return, laboratory tests) | ☑ p. 13-14 |
| | 11d | Relevant concomitant care and interventions that are permitted or prohibited during the trial | ☑ p. 6 |
| Outcomes | 12 | Primary, secondary, and other outcomes, including the specific measurement variable (eg, systolic blood pressure), analysis metric (eg, change from baseline, final value, time to event), method of aggregation (eg, median, proportion), and time point for each outcome. Explanation of the clinical relevance of chosen efficacy and harm outcomes is strongly recommended | ☑ p. 12-13 |
| Participant timeline | 13 | Time schedule of enrolment, interventions (including any run-ins and washouts), assessments, and visits for participants. A schematic diagram is highly recommended (see Figure) | ☑ p. 6-7, 14 |
| Sample size | 14 | Estimated number of participants needed to achieve study objectives and how it was determined, including clinical and statistical assumptions supporting any sample size calculations | ☑ p. 7 |
| Recruitment | 15 | Strategies for achieving adequate participant enrolment to reach target sample size | ☑ p. 5-6 |


For peer review only - http://bmjopen.bmj.com/site/about/guidelines.xhtml

**Methods: Assignment of interventions (for controlled trials)**

Allocation:

| | | | |
|---|---|---|---|
| Sequence generation | 16a | Method of generating the allocation sequence (eg, computer-generated random numbers), and list of any factors for stratification. To reduce predictability of a random sequence, details of any planned restriction (eg, blocking) should be provided in a separate document that is unavailable to those who enrol participants or assign interventions | ☑ p. 8 |
| Allocation concealment mechanism | 16b | Mechanism of implementing the allocation sequence (eg, central telephone; sequentially numbered, opaque, sealed envelopes), describing any steps to conceal the sequence until interventions are assigned | ☑ p. 8 |
| Implementation | 16c | Who will generate the allocation sequence, who will enrol participants, and who will assign participants to interventions | ☑ p. 8 |
| Blinding (masking) | 17a | Who will be blinded after assignment to interventions (eg, trial participants, care providers, outcome assessors, data analysts), and how | ☑ p. 8 |
| | 17b | If blinded, circumstances under which unblinding is permissible, and procedure for revealing a participant's allocated intervention during the trial | N/A |

**Methods: Data collection, management, and analysis**

| | | | |
|---|---|---|---|
| Data collection methods | 18a | Plans for assessment and collection of outcome, baseline, and other trial data, including any related processes to promote data quality (eg, duplicate measurements, training of assessors) and a description of study instruments (eg, questionnaires, laboratory tests) along with their reliability and validity, if known. Reference to where data collection forms can be found, if not in the protocol | ☑ p. 12-13 |
| | 18b | Plans to promote participant retention and complete follow-up, including list of any outcome data to be collected for participants who discontinue or deviate from intervention protocols | ☑ p. 14 |
| Data management | 19 | Plans for data entry, coding, security, and storage, including any related processes to promote data quality (eg, double data entry; range checks for data values). Reference to where details of data management procedures can be found, if not in the protocol | ☑ p. 16 |
| Statistical methods | 20a | Statistical methods for analysing primary and secondary outcomes. Reference to where other details of the statistical analysis plan can be found, if not in the protocol | ☑ p. 14-15 |



For peer review only - http://bmjopen.bmj.com/site/about/guidelines.xhtml

| | | | |
|---|---|---|---|
| | 20b | Methods for any additional analyses (eg, subgroup and adjusted analyses) | ☑ p. 14-15 |
| | 20c | Definition of analysis population relating to protocol non-adherence (eg, as randomised analysis), and any statistical methods to handle missing data (eg, multiple imputation) | ☑ p. 14-15 |
| **Methods: Monitoring** | | | |
| Data monitoring | 21a | Composition of data monitoring committee (DMC); summary of its role and reporting structure; statement of whether it is independent from the sponsor and competing interests; and reference to where further details about its charter can be found, if not in the protocol. Alternatively, an explanation of why a DMC is not needed | ☑ p. 16 |
| | 21b | Description of any interim analyses and stopping guidelines, including who will have access to these interim results and make the final decision to terminate the trial | N/A |
| Harms | 22 | Plans for collecting, assessing, reporting, and managing solicited and spontaneously reported adverse events and other unintended effects of trial interventions or trial conduct | ☑ p. 15 |
| Auditing | 23 | Frequency and procedures for auditing trial conduct, if any, and whether the process will be independent from investigators and the sponsor | ☑ p. 16 |
| **Ethics and dissemination** | | | |
| Research ethics approval | 24 | Plans for seeking research ethics committee/institutional review board (REC/IRB) approval | ☑ p. 16 |
| Protocol amendments | 25 | Plans for communicating important protocol modifications (eg, changes to eligibility criteria, outcomes, analyses) to relevant parties (eg, investigators, REC/IRBs, trial participants, trial registries, journals, regulators) | ☑ p. 16 |
| Consent or assent | 26a | Who will obtain informed consent or assent from potential trial participants or authorised surrogates, and how (see Item 32) | ☑ p. 7 |
| | 26b | Additional consent provisions for collection and use of participant data and biological specimens in ancillary studies, if applicable | N/A |
| Confidentiality | 27 | How personal information about potential and enrolled participants will be collected, shared, and maintained in order to protect confidentiality before, during, and after the trial | ☑ p. 16 |
| Declaration of interests | 28 | Financial and other competing interests for principal investigators for the overall trial and each study site | ☑ p. 17 |

For peer review only

| | | | |
|---|---|---|---|
| Access to data | 29 | Statement of who will have access to the final trial dataset, and disclosure of contractual agreements that limit such access for investigators | ☑ p. 16 |
| Ancillary and post-trial care | 30 | Provisions, if any, for ancillary and post-trial care, and for compensation to those who suffer harm from trial participation | N/A |
| Dissemination policy | 31a | Plans for investigators and sponsor to communicate trial results to participants, healthcare professionals, the public, and other relevant groups (eg, via publication, reporting in results databases, or other data sharing arrangements), including any publication restrictions | ☑ p. 16 |
| | 31b | Authorship eligibility guidelines and any intended use of professional writers | ☑ p. 16 |
| | 31c | Plans, if any, for granting public access to the full protocol, participant-level dataset, and statistical code | N/A |
| **Appendices** | | | |
| Informed consent materials | 32 | Model consent form and other related documentation given to participants and authorised surrogates | ☑ |
| Biological specimens | 33 | Plans for collection, laboratory evaluation, and storage of biological specimens for genetic or molecular analysis in the current trial and for future use in ancillary studies, if applicable | N/A |

*It is strongly recommended that this checklist be read in conjunction with the SPIRIT 2013 Explanation & Elaboration for important clarification on the items. Amendments to the protocol should be tracked and dated. The SPIRIT checklist is copyrighted by the SPIRIT Group under the Creative Commons "Attribution-NonCommercial-NoDerivs 3.0 Unported" license.

For peer review only

BMJ Open

# Testing the effectiveness of a weight loss intervention to enhance self-regulation in adults who are obese: protocol for a randomised controlled trial

| | |
|---|---|
| Journal: | *BMJ Open* |
| Manuscript ID | bmjopen-2019-031572.R1 |
| Article Type: | Protocol |
| Date Submitted by the Author: | 26-Oct-2019 |
| Complete List of Authors: | Frie, Kerstin; University of Oxford, Nuffield Department of Primary Care Health Sciences<br>Hartmann-Boyce, Jamie; University of Oxford, Nuffield Department of Primary Care Health Sciences<br>Jebb, Susan; University of Oxford, Nuffield Department of Primary Care Health Sciences<br>Aveyard, Paul; University of Oxford, Nuffield Department of Primary Care Health Sciences |
| <b>Primary Subject Heading</b>: | Public health |
| Secondary Subject Heading: | Nutrition and metabolism |
| Keywords: | self-regulation, self-monitoring, self-experimentation, weight loss, obesity |
| | |

SCHOLARONE™
Manuscripts

# Testing the effectiveness of a weight loss intervention to enhance self-regulation in adults who are obese: protocol for a randomised controlled trial

Kerstin Frie[1], Jamie Hartmann-Boyce[1], Susan A Jebb[1], Paul Aveyard[1]

[1] Nuffield Department of Primary Care Health Sciences, University of Oxford, UK

Corresponding Author: Kerstin Frie
E: kerstin.frie@phc.ox.ac.uk, T: +44 (0) 1865 289317, Postal address:
Nuffield Department of Primary Care Health Sciences, University of Oxford, Radcliffe Primary Care Building, Radcliffe Observatory Quarter, Woodstock Road, Oxford, OX2 6GG

Word count: 4092

For peer review only

## ABSTRACT

**Introduction:** Previous trials finding an effect of self-monitoring on weight loss have considered the effect to be mediated by self-regulatory processes. However, a qualitative think-aloud study asking people to record thoughts and feelings during weighing showed that self-regulation occurs only rarely without further instruction. The aim of this trial is to test a novel intervention guiding people through the self-regulatory processes to see whether it facilitates weight loss.

**Methods and analyses:** A parallel group, randomised controlled trial will be conducted to test the concept that a self-regulation intervention for weight loss increases weight loss compared to daily self-weighing without further support. One hundred participants with a BMI≥30 kg/m$^2$ will be randomised to either the control or intervention group. The control group will be asked to weigh themselves daily for eight weeks, the intervention group will be encouraged to follow the self-regulation intervention. They will be prompted to weigh daily, track their weight using an app, plan daily actions for weight loss and reflect on their action plans on a weekly basis. This self-regulation cycle will allow them to experiment with different weight loss strategies and identify effective and sustainable actions. Primary and process outcomes will be measured at baseline and 8-weeks follow-up. Linear regression analysis of the primary outcome, weight change, will assess the early effectiveness of the intervention. The process outcomes liking, perceived effectiveness, as well as usage and barriers with regards to the self-regulation intervention, will be assessed through qualitative analysis of follow-up interviews and quantitative analysis of adherence rates and responses to a final questionnaire.

**Ethics and dissemination:** This trial was reviewed and approved by the NHS National Research Ethics Committee (REC) and the Health Research Authority (HRA, reference number: 18/SC/0482). The findings of the trial will be published in peer reviewed journals and presented at conferences.



**Trial registration number:** ISRCTN14148239, Pre-recruitment.

**Protocol Version:** Version 1.1, 7th of December 2018

**Keywords:** self-regulation, self-monitoring, self-experimentation, weight loss, obesity




For peer review only - http://bmjopen.bmj.com/site/about/guidelines.xhtml

## STRENGTHS AND LIMITATIONS OF THIS STUDY

**Strengths**

- This trial will provide preliminary evidence for whether a remotely delivered and iterative self-regulation intervention can enhance weight loss
- The intervention addresses specific barriers to self-regulation, identified in a previous qualitative study of experiences of self-weighing
- Process evaluation measures will assess participants liking, usage and perceived effectiveness of the intervention to enable future improvements

**Limitations**

- Blinding might be compromised as participants might be aware that they were assigned to the control or treatment group
- The study is designed as a 'proof-of-concept' trial and longer-term studies will need to evaluate the effectiveness of the intervention as a weight management tool

For peer review only



For peer review only - http://bmjopen.bmj.com/site/about/guidelines.xhtml

## INTRODUCTION

Self-monitoring of weight is often employed in multi-component weight loss interventions, as evidence suggests it aids weight loss(1-5). The weight loss effect has often been ascribed to a self-regulation mechanism, based on the hypothesis that self-monitoring triggers self-regulation(1, 4-6). In this context, self-regulation occurs in iterative cycles, starting by (1) contextualising the weight with previous measurements and goals, thus providing (2) an opportunity to reflect on previous behaviour and reinforce successful actions, enabling (3) the planning of actions to reach the goal, followed by (4) the performance of planned actions(6-8). This cycle of processes allows for experimentation with different weight loss techniques, helping the user to build a personal portfolio of effective and sustainable strategies(9, 10).

A study employing self-weighing as a standalone intervention did not find a significant weight loss effect, raising the question whether self-regulation is performed naturally after weighing or whether additional weight loss treatment components are necessary(11). We addressed this question in a think-aloud study, where twenty-four participants were asked to record their thoughts and feelings during daily weighing for eight weeks, without being prompted to self-regulate(12). On 90% of occasions, participants contextualised their weight measurement and on 58% participants reflected on previous behaviours. Only on 20% of occasions did participants plan actions and specific action planning, defining a concrete action and time plan, was rare (6%). The frequency of specific action planning was, however, significantly predictive of weight loss. Hence, the study provided support to the notion that completing the last step of the self-regulation process can elicit weight loss. However, the think-aloud study also showed that self-regulation does not occur autonomously, and that people need support in developing self-regulation skills, especially action planning. Self-regulation, once learnt, has the potential to be a weight loss strategy that is performed autonomously and sustainably. It provides an opportunity for remote weight loss interventions that do not require resource-intensive support from health care providers.

Several weight loss interventions including self-regulation components have been developed over the years. Some educate their users about self-regulation theory without providing active support for the key component - specific action planning(1, 4, 13, 14). Others support users in action planning, but fail to imitate the iterative nature of self-regulation, as they do not allow the usage of self-monitoring feedback for adaptations to the action plans(15-18), thus disabling self-regulation. Some interventions guide participants through iterative reformulation of action plans following self-monitoring feedback, but are delivered through face-to-face sessions(19, 20), which are resource-intensive and not easily rolled out at large scale. Other interventions incorporating action



For peer review only - http://bmjopen.bmj.com/site/about/guidelines.xhtml

planning dictate which actions participants are supposed to follow(15, 21, 22), which might reduce goal ownership, a significant predictor of goal engagement and attrition(23, 24). Notwithstanding these critiques, seven of the eleven studies cited here found significant weight loss, suggesting that components targeting the self-regulation cycle can enhance weight loss, encouraging further work in this area. Our critiques highlight the need for an intervention that guides participants through the whole and iterative self-regulation process in an autonomous, low-cost and scalable manner. Furthermore, since many interventions add self-regulation elements to a broader spectrum of weight loss treatment components(14, 17, 25-27), a study testing self-regulation as a standalone intervention is needed to investigate whether iterative self-regulation is sufficient to achieve weight loss.

With this study we aim to test the proof of concept of an intervention aiming to address this gap in the literature. The PREVAIL intervention (**P**eople **RE**gulating themsel**V**es to **A**chieve we**I**ght **L**oss) is a weight loss programme guiding people through the iterative self-regulation process. It encourages users to experiment with different weight loss approaches, and use the self-regulation mechanism to find their ideal set of tools.

## OBJECTIVES

The primary objective of this trial is to test the concept whether an intervention, which trains individuals in self-regulatory processes, aids early weight loss in comparison to unsupported daily weighing. Other objectives pertain to the evaluation of usage and effectiveness of the self-regulation intervention components, as well as the qualitative analysis of participant experiences of the intervention.

## METHODS

### Study design and setting

An individually randomised, two arm, parallel group design will be employed, assessing superiority of the self-regulation intervention over daily self-weighing alone. Participation will last eight weeks. This length was deemed sufficient to assess early effectiveness of the intervention, as previous studies have been able to detect weight loss effects after two months(28). If the results are promising, the data will provide good evidence to justify conducting a longer-term randomised controlled trial. Participants will attend two study visits, one at baseline and one after the end of the 8th week of the intervention. The primary outcome will be weight change. The study will take place in Oxfordshire, UK and run between April to October 2019.



For peer review only - http://bmjopen.bmj.com/site/about/guidelines.xhtml

**Recruitment**

Two to four general practitioner (GP) practices around Oxford, UK, will function as participant identification centres and search their health records to identify suitable patients for the trial (age ≥ 18 years, BMI ≥30kg/m$^2$). The GP will screen the search list and exclude patients who would be inappropriate to invite, including terminally ill or violent patients. Suitable patients will be sent an invitation letter from their GP. They will be encouraged to contact the research team if they are interested in taking part. GPs may also identify suitable patients during routine consultations. We will ask practices not to refer participants to commercial weight loss programmes, other obesity clinics or bariatric surgery, whilst they are enrolled as participants in this trial.

**Eligibility criteria**

Inclusion Criteria

- Participant is willing and able to give informed consent
- Aged 18 years or above
- BMI ≥30 kg/m$^2$
- Owns an Apple or Android smartphone

Exclusion Criteria

The participant may not enter the study if any of the following apply:

- Unable to understand English
- Unable to follow all intervention procedures for a period of more than 4 consecutive days
- Currently self-monitoring body weight more than once a week
- Currently or within three months of study entry attended a weight management programme or currently participating in another weight loss study
- Lost more than 5% of current body weight in the last six months
- Prior bariatric surgery, or scheduled for bariatric surgery
- Pregnant, or planning to become pregnant during the course of the study
- Have an electronic medical implant, such as a pacemaker
- Have ever had or been diagnosed with an eating disorder
- People that the GP judges not able to meet the demands of either treatment programme or measurement schedule. This may include severe medical problems not listed above.



For peer review only - http://bmjopen.bmj.com/site/about/guidelines.xhtml

**Participant flow**

Screening

People who are interested in taking part will contact the research team. The research team will then discuss study participation by telephone or email and undertake screening. If the person appears eligible and would like to attend a baseline visit, the research team will offer an appointment at a local venue. The participants will be emailed a participant information sheet (PIS).

Baseline

When the participants attend the baseline appointment, a member of the research team will seek informed consent and check eligibility for inclusion in the study by measuring height and weight for BMI calculation. The participants will be asked to complete an online questionnaire, capturing demographics (i.e., age, gender, ethnicity) and previous experiences with self-weighing. Participants will be randomised and receive instructions for the assigned intervention. The researcher will provide participants with a body scale for the duration of the trial. A follow-up appointment will be scheduled for after the completion of the intervention period.

Follow up

The aim of the follow-up appointment is to assess the outcomes of the trial. The research team will email participants in advance to remind them of the meeting. In the intervention group, this email will also contain a final questionnaire, asking participants about the usefulness of each of the intervention components and an overall rating of the intervention.

The appointment will be scheduled at a local venue and conducted by a member of the research team. Participants will be asked to return the body scales. The researcher will measure participants' weight.

Twenty participants in the intervention group will be invited to participate in a semi-structured interview at the end of the follow-up meeting. The interviewees will be sampled by the lead researcher with the aim to reflect different levels of adherence, weight change, and responses to the final questionnaire. Participants will be asked about their experiences with self-weighing, their liking of the intervention components, as well as perceived barriers to engaging with the intervention. Interviews will be recorded, transcribed and analysed. A study flow chart is displayed in Figure 1.

| Insert Figure 1 here |
|---|
| Figure 1: Study Flow Chart |



For peer review only - http://bmjopen.bmj.com/site/about/guidelines.xhtml

**Sample size**

We intend to recruit 100 participants, which is sufficient to detect a 1.5kg difference between conditions, at 90% power and 5% type I error rate, while allowing for a 20% drop-out rate. Variance of weight change was based on results of a similar trial(28), which reported a standard deviation of 2.13kg at 2 months follow-up.

**Randomisation and blinding**

All eligible participants will be randomised with an allocation ratio of 1:1 to the intervention or control group. A randomisation sequence, stratified by GP and using block randomisation with randomly varying block sizes of 2 and 4 will be generated using a computer algorithm. Allocations will be concealed in numbered, sealed, opaque envelopes by an independent researcher in the department and handed to the researcher who will conduct the baseline visits. Due to the nature of this trial, it will be difficult to blind participants to the treatment allocation. We will aim to make it as opaque as possible by presenting daily weighing as an intervention to control group participants, as done in previous trials(29). The researchers conducting baseline and follow-up will perform data analysis, and will not be blinded to treatment allocation. The primary outcome, weight change, will be measured objectively. Adherence to self-regulation steps will be measured objectively through the frequency of weight logs and completed questionnaires in control and intervention group. The evaluations of treatment components in the final questionnaire will be measured without researchers' input and analysed quantitatively. Blinding of the researcher who conducts and analyses the semi-structured interviews with intervention group participants will not be possible.

**Intervention and control**

Intervention

Participants will be asked to weigh themselves every morning after waking using standard body scales (Etekcity Corporation, California) provided to them. In addition, they will be prompted to complete tasks which speak to the different steps of the self-regulation process, including (1) contextualising the weight measurement, (2) reflecting on behaviour, and (3) planning weight loss actions. The individual tasks and their development are described in more detail below. Input from members of the public was sought at several stages of the intervention development.

(1) Contextualising: In the think-aloud study participants struggled memorising daily measurements and keeping an overview of their weight loss progress, which impeded their ability to use weight measurements as constructive feedback. Participants stated they would have benefitted from a weight-tracking tool. Therefore, to support participants in contextualising their weight



For peer review only - http://bmjopen.bmj.com/site/about/guidelines.xhtml

measurements, we will encourage them to use the app "Weight Loss Tracker, BMI" by aktiWir GmbH. Research shows that digital tracking devices can significantly increase adherence to self-monitoring(30, 31), perhaps because the visualization of progress and feedback on weight loss success provides motivation and keeps users on track with their goals(32).

(2) Reflecting: The think-aloud study showed that participants struggled to interpret day-to-day weight changes due to daily fluctuations that were not caused by fat loss or gain(12). We therefore decided to encourage reflection on a weekly rather than daily basis. Participants will receive weekly emails with feedback on their weekly weight trends, asking them to complete an online questionnaire (Qualtrics, USA). The questionnaire will prompt reflection on the relationship between behaviours performed throughout the week and weight change observed. Participants will be asked to use this insight to evaluate the use of the weight loss actions they had performed throughout the week.

(3) Planning: We aimed to strike a balance between ensuring that participants choose appropriate actions and allowing them to choose actions themselves, as lack of goal ownership predicts attrition(23). Participants will therefore choose one weight loss action per day from a list of 53 actions (see Appendix 1). To create the actions list, we first identified weight loss actions from effective weight loss interventions in the literature. These were reviewed, adapted and complemented during iterative brainstorming sessions with an interdisciplinary expert team, comprised of dietitians, general practitioners and psychologists.

The rationale for daily action plans was twofold: 1) allowing participants to adapt their actions flexibly to their day and 2) giving participants exposure to a wider range of strategies.

Based on action planning and implementation intention research, which shows that the specificity of action plans increases likelihood of implementation(33-35), we will ask participants to specify where, when and how they will perform their chosen action, which cues they will use, which barriers they might experience and how they will deal with them.

As participants only reflect on their behaviour on a weekly basis, we grouped the actions conceptually into seven categories, five of which cover diet-related actions and two of which cover physical activity-related actions. Participants will be asked to choose one category at the beginning of the week, and choose daily actions from this category for the rest of the week. Weekly behaviour reflection will therefore focus on the effectiveness of a category of actions. Taken together, the reflection and action planning process shall enable participants to experiment with different weight loss approaches and decide on their effectiveness and usefulness based on trends in weight data.

At the beginning of weeks two through eight of the intervention, participants will be prompted to commit to some of the actions of previous weeks, as the performance of several weight loss actions increases chances for weight loss.



For peer review only - http://bmjopen.bmj.com/site/about/guidelines.xhtml

Action planning support will be provided through daily questionnaires (Qualtrics, USA), which will be sent to participants every morning via email.

At baseline, participants will receive detailed instructions about the intervention with the aid of a manual which they may take home afterwards. The manual will explain the rationale of self-regulation and experimentation to participants and give tips on how to best weigh on a daily basis. It will also provide information on the different weight loss actions participants can try throughout the study.

Participants will also be given an action diary, in which they will be asked to record their performed weight loss actions.

We will call participants at the end of the 1st and 4th week to ask about and solve any technical problems that may have arisen (e.g. with the functioning of the scales or the receiving of questionnaires). A figure from the study manual depicting the intervention procedure is displayed in Figure 2. The TiDIER checklist(36) for the PREVAIL intervention and the comparator is reported in Table 1.

Insert Figure 2 here

Figure 2: Intervention procedure as depicted in study manual

Table 1: TiDIER checklist describing the intervention and control condition

| | Intervention: PREVAIL | Control: Daily Self-Weighing without behavioural support |
|---|---|---|
| BRIEF NAME | PREVAIL (**P**eople **Re**gulating Themsel**v**es to **A**chieve We**i**ght **L**oss) | Self-Weighing Only |
| WHY | **Self-weighing:** Monitoring weight on a daily basis will enable participants to take note of their weight loss progress.<br>**Weight-Tracking:** Our preceding study(12) found that people can lose track of their weight loss progress when weighing every day because they struggle to remember measurements. We therefore ask participants to track their weight.<br>**Action Planning:** In our preceding study, participants rarely made action plans to help them progress with their weight loss(12). If they did make action plans, they were rarely specific. Specificity of action plans is a significant predictor of the likelihood of implementation(33-35). We therefore guide participants through a specific action planning process.<br>**Report Email:** Participants in our preceding study struggled to see trends in their weight data(12). Unfortunately, the app we are using for weight-tracking does not provide users with trend information. We will therefore send out weekly emails, containing a statement about the trend of the weight measurements of the last week.<br>**Reflection and Action Evaluation:** Our preceding study revealed that daily fluctuations vary over time within people, making it difficult to interpret daily weight changes. We therefore want to encourage participants to reflect on their weight changes and the effectiveness of their weight loss | A previous trial has found that self-monitoring of weight without further guidance is not effective for weight loss.(11) |

For peer review only



For peer review only - http://bmjopen.bmj.com/site/about/guidelines.xhtml

| | | |
|---|---|---|
| | actions on a weekly basis. Using the weekly weight trend information, participants will be able to evaluate whether they found the group of actions they performed effective and worth repeating. | |
| WHAT | **Self-weighing:** Participants will be instructed to weigh themselves daily using simple digital body scales (Etekcity Corporation, California). They will be asked to weigh themselves in a similar state every day, ideally first thing in the morning and without clothes.<br>**Weight-Tracking:** Participants will be asked to download the free app "Weight Loss Tracker, BMI" by aktiWir GmbH on their smartphone and use it on a daily basis to record their weight measurements. They will be asked to submit a backup of their data to the research team every week.<br>**Action Planning:** Participants will receive a daily questionnaire helping them to plan a weight loss action. The questionnaire will start by asking participants to enter their morning weight. At the beginning of first week they will then be asked to choose a category of actions, and they will be able to choose one of the actions within this category per day for the rest of the week. There are seven categories in total, five covering diet related actions and two covering physical activity related actions. Participants will be asked to specify for each action how, when, and where they are going to perform it, and which cues they are going to use. They will also be prompted to think about how to overcome potential barriers. At the start of weeks 2 through 8, participants will be encouraged to try out a new category of actions. We will additionally ask participants on a weekly basis to commit to continuing some of the actions they tried out in previous weeks. In order to help participants to maintain an overview of the actions they performed so far, we will provide them with a non-digital action plan diary.<br>**Report Email:** Once a week, participants will receive an email from the research team, informing them about their trend weight change for the last week. This trend weight change consists of the slope of a trend line fitted across all measurements of the week, multiplied by the number of days covered by the measurements. The weight measurements used to create these reports will be taken from the daily action planning questionnaires.<br>**Reflection and Action Evaluation:** In the weekly report email, participants will receive a link to the reflection and action evaluation questionnaire. This questionnaire prompts participants to think about why their weight has changed as it has. They are further asked to evaluate the group of actions they performed across the week, including whether they found them useful and whether they would repeat them. On the basis of this evaluation, participants will be able to decide which actions they want to continue doing in the next weeks, and which ones to drop. Using this method of self-experimentation, participants will be able to try and test different weight loss strategies and identify the ones that are effective and sustainable for them. | Self-weigh every morning in a similar state, using smart scales (BodyTrace, Inc., New York). |

For peer review only

| | | |
|---|---|---|
| WHO | The chief investigator (KF) will deliver the intervention at the baseline session. She will also organise the mailing of all questionnaires and weekly report emails. She will be the primary contact for all participants. KF is a psychologist by background and has received GCP training. | The chief investigator (KF) will instruct participants in the control group to weigh themselves every day. |
| HOW | Participants will measure their weight on provided body scales. They will use a free weight-tracking app called "Weight Loss Tracker, BMI" by aktiWir GmbH on their smartphone to track their weight loss progress. All questionnaires will be sent automatically to participants in the early morning by the survey platform Qualtrics (USA). | Participants measure their weight on provided body scales. |
| WHERE | At home. | At home. |
| TAILORED | Participants are able to tailor the intervention to themselves by choosing action plans relevant to them. | N/A |
| HOW Well | We will request participants to send us a back-up of their data in the weight-tracking app. This data will allow us to assess adherence to weight-tracking. Completion of the daily action planning and weekly reflection questionnaires will allow us to assess adherence to action planning and reflection. Adherence to daily weighing will be assessed by combining weight records from the weight tracking app and action planning questionnaires. | The provided body scales contain a SIM card which automatically transfers the weight data to a secure research server. We will therefore be able to assess adherence. |

Control

Participants in the control group will be instructed to self-weigh daily and see what daily weighing motivates them to do. They will not receive any further instructions. By using this comparator group, we want to test whether the self-regulation process can enhance self-weighing to be an effective weight loss tool. Participants will receive smart scales (BodyTrace, Inc., New York) which are equipped with a SIM card and automatically transfer measurements to a secure server via the 3G/4G network. This will allow us to assess adherence to daily weighing in the control group.

**Patients and public involvement (PPI)**

Members of the public were involved in the design of the study at several stages. After creating the invitation letter, PIS, informed consent form, semi-structured interview guide, as well as the baseline, daily, weekly and follow-up questionnaires for the PREVAIL intervention, we asked members of the public for feedback. Our department has a panel of >100 members of the public with an interest in weight management. Based on phone calls with members of this panel we were able to make the materials clearer and more concise. The manual, explaining the intervention in detail, was further discussed with members of the panel in a focus group session. As a result of this focus group session, the manual was professionally edited to be shorter. We also added figures and graphs to present the procedures of the study more visually. The panel helped to revise the explanations of the different action plans.



For peer review only - http://bmjopen.bmj.com/site/about/guidelines.xhtml

A test run of four weeks was conducted with five members of the department, who are not otherwise involved in this study. They provided feedback on the running of the intervention, which, amongst other outcomes, led to the creation of a reminder email which will be sent out before the start of the intervention. No members of the public will be involved in conducting or analysing the study. However, we will gather input on the most suitable and effective ways to disseminate our research findings to the public.

**Outcomes**

Primary outcome

- Change in body weight between baseline and follow-up by condition

Secondary outcomes (Process evaluation)

- Adherence to self-regulation steps, assessed through weight records and daily action planning/weekly evaluation questionnaires
- Test moderators of effectiveness: adherence measures, highest educational qualification, liking of weighing at baseline, overall rating of intervention in final questionnaire
- Perceived effectiveness of intervention and liking of intervention features based on final questionnaire and follow-up interviews
- Barriers and unmet needs for successful weight loss, assessed in follow-up interviews

**Measurements**

A schedule of measurements can be found in Table 2.

Physical measurements

Participants' height will be measured at baseline to the nearest 0.1cm using a stadiometer. Weight will be measured both at baseline and follow-up using a digital scale (SC-240 MA, Tanita Japan). Weight will be recorded to the nearest 0.1kg.

Process Evaluation Measures

*Adherence measures*

For the control group, adherence to daily weighing will be measured by calculating the proportion of days for which we have a recorded weight measurement on the BodyTrace server.

For the intervention group, adherence to daily weighing will be assessed by calculating the proportion of days for which a weight was recorded in the weight-tracking app or in the daily action planning questionnaire. Adherence to weight-tracking will be calculated as the proportion of days for which a weight measurement was recorded in the app. Adherence to action planning and

For peer review only

reflection will be measured by calculating the proportion of days on which the respective questionnaires were completed. An overall adherence score will be calculated averaging adherence rates across all intervention components.

*Evaluation of intervention components*

Using the data from the final online questionnaire, we will calculate means and standard deviations of the ratings for each intervention component.

Table 2: Schedule of measurements.

| | Screening | Baseline visit | Intervention Period | Completion email | Follow-up visit (after 8 weeks) |
|---|---|---|---|---|---|
| Length | 10 mins | Up to 1h | 10 mins per day | 5 mins | up to 45 mins |
| Who conducts | Research Team | Research Team | Participants | Research Team | Research Team |
| Eligibility assessment | ☑ | ☑ (BMI) | | | |
| Enrolment | | ☑ | | | |
| Baseline questionnaire | | ☑ | | | |
| Weight | | ☑ | ☑ | | ☑ |
| Height | | ☑ | | | |
| Allocation | | ☑ | | | |
| Weight-tracking (only intervention) | | | ☑ | | |
| Daily Questionnaire (only intervention) | | | ☑ | | |
| Weekly report and questionnaire (only intervention) | | | ☑ | | |
| Final questionnaire (only intervention) | | | | ☑ | |
| Semi-structured interview (20 participants in intervention group) | | | | | ☑ |

**Retention**

The daily questionnaire and weekly report emails will act as prompts for the participants to engage with the intervention. At the end of the follow-up visit, participants will receive a £35 one4all gift card.

There are no criteria for withdrawal other than participants’ request to withdraw. Participants can also ask to withdraw their collected data. We will ask participants wishing to withdraw whether they are willing to attend the final follow-up and take part in an exit interview to understand the reasons for their discontinuation.



For peer review only - http://bmjopen.bmj.com/site/about/guidelines.xhtml

For peer review only

**Statistical analyses**

The statistical analysis of the primary outcome, effectiveness of the intervention for weight loss, will be carried out on the basis of intention-to-treat (ITT). That is, after randomisation, participants will be analysed according to their allocated intervention group. We will endeavour to obtain full follow-up data on every participant to allow full ITT analysis. A linear regression, predicting weight at 8-weeks follow-up while adjusting for baseline weight and GP practice, will assess the effect of condition(37). We will assess the sensitivity of the analysis to different assumptions about missing data using a variety of imputation methods, including baseline observation carried forward analysis and an analysis of participants completing follow-up. A final analysis will impute the last home-measured weight for people who did not attend the final follow-up. All tests will be done at a 5% two-sided significance level.

Secondary outcomes will be analysed in several ways. Adherence rates will be assessed and compared between the experimental and control condition. Moderator analyses will assess the effect of adherence, highest educational qualification, liking of weighing at baseline, and overall intervention rating at follow-up (only intervention condition) on weight change.

Further exploratory analyses may be added post-hoc based on preliminary findings. The statistical analysis plan can be found in Appendix 2.

**Qualitative study**

All interview audio-recordings will be transcribed and entered into the NVivo software package (QSR International) for qualitative data analysis. Framework analysis according to Ritchie and Spencer(38) will assess the participants' experiences and perceptions of the different intervention components. The findings will be put into context with the results from the final questionnaire. Inductive thematic analysis following Braun and Clarke(39) will explore additional themes, including barriers and unmet needs. One researcher with training in qualitative methods will perform coding for all interviews.

**Trial management group**

The day-to-day management and operation of the study will be coordinated by KF. A Trial Management Group (TMG), consisting of the authors of this paper, will have oversight of the trial. The TMG will be responsible for the monitoring of all aspects of the trial's conduct and progress and will ensure that the protocol is adhered to and that appropriate action is taken to safeguard participants and the quality of the trial itself. The TMG will meet regularly throughout the course of the trial.



For peer review only - http://bmjopen.bmj.com/site/about/guidelines.xhtml

**Adverse events**

This is a low risk trial where it is implausible that the intervention will lead to differences in the occurrence of adverse events so we decided that it was inappropriate to burden participants to collect and record these.

**Trial monitoring**

This is a short trial with no adverse event monitoring or stopping rules so we deemed that a trial steering committee and a data monitoring committee were unnecessary.

**Data management**

Data will be kept in accordance with GCP, the Data Protection Act 2018 (DPA) and General Data Protection Regulation (GDPR). Two separate databases will be created, one containing all participant identifiable information, the other capturing all outcome data in an anonymised manner, using a unique participant ID. Weight, height and body composition measurements will be entered into the second database by the researcher. Data from the online questionnaires will be downloaded from Qualtrics and added to the second database. The two databases as well as the anonymised recordings and transcriptions from follow-up interviews will be stored on the secure departmental drive and will only be accessible by members of the TMG. After a lay summary of results has been sent out to participants, the database with participant identifiable data will be destroyed. We will retain the anonymised database for future secondary analyses.

Direct access to study data will be granted to authorised representatives from the sponsor for monitoring and/or audit of the study to ensure compliance with regulations. This access, the reason for it and who has authorised it will be recorded by the TMG. Otherwise, confidentiality will be maintained and no-one outside the TMG will have access to the database.

**Ethics and dissemination**

This trial was reviewed and approved by the NHS National Research Ethics Committee (REC) and the Health Research Authority (HRA, reference number: 18/SC/0482). Any substantial changes to the protocol will be submitted as an amendment to these institutions, as well as the Sponsor. Upon completion of the trial, KF will submit an End of Study notification and final report to the REC Committee, HRA, and Sponsor.

We intend to publish the results of this study in peer reviewed journals, regardless of the nature of the outcome. Authorship will be determined in accordance with the ICMJE guidelines. We will also present our findings at national and international conferences, and publicise our publications through the departments' online presence. Participants will be informed of the trial



For peer review only - http://bmjopen.bmj.com/site/about/guidelines.xhtml

results through an information sheet prepared for a lay audience. We will also inform our PPI panel members about the findings of the study through their regular newsletter.

**Acknowledgements**

We would like to acknowledge and thank the members of the public who have helped design the PREVAIL intervention. We thank members of the department who have contributed in expert brainstorming sessions to the development of the intervention. We would like to thank Carmen Piernas and Rhiannon Edwards for creating the randomisation sequence and the randomisation envelopes. We thank the Clinical Trials Unit of the Nuffield Department of Primary Care Health Sciences for their help with setting up this study and support in conducting the trial.

**Author Contributions**

KF, JHB, SJ, and PA contributed to the design of the intervention and this study. KF led the preparation of the trial. All authors commented and worked on this paper.

**Funding**

This research is funded by the National Institute for Health Research (NIHR) Collaboration for Leadership in Applied Health Research and Care Oxford (CLAHRC) at Oxford Health NHS Foundation Trust. KF's time on this project is funded by NIHR CLAHRC Oxford at Oxford Health NHS Foundation Trust, Wolfson College, University of Oxford (Oxford-Wolfson Marriott-Primary Care Graduate Scholarship), and NIHR School for Primary Care Research (NIHR SPCR). JHB's, SJ's and PA's time on this project is funded by the NIHR Oxford Biomedical Research Centre (BRC) and Oxford CLAHRC. PA and SJ are NIHR senior investigators. The views expressed are those of the authors and not necessarily those of the NHS, the NIHR, the Wellcome Trust, or the Department of Health and Social Care.

**Sponsor**

University of Oxford
University Offices
Wellington Square
Oxford
OX1 2JD
United Kingdom

The sponsor has reviewed all participant-facing documents as part of the ethics application. The sponsor is not involved in the collection, management, analysis,



For peer review only - http://bmjopen.bmj.com/site/about/guidelines.xhtml

and interpretation of data; writing of the report; and the decision to submit the report for publication of the study.

**Competing interests statement**

The authors have no known competing interests to declare.

**Licence Statement**

I, the Submitting Author has the right to grant and does grant on behalf of all authors of the Work (as defined in the below author licence), an exclusive licence and/or a non-exclusive licence for contributions from authors who are: i) UK Crown employees; ii) where BMJ has agreed a CC-BY licence shall apply, and/or iii) in accordance with the terms applicable for US Federal Government officers or employees acting as part of their official duties; on a worldwide, perpetual, irrevocable, royalty-free basis to BMJ Publishing Group Ltd ("BMJ") its licensees and where the relevant Journal is co-owned by BMJ to the co-owners of the Journal, to publish the Work in BMJ Open and any other BMJ products and to exploit all rights, as set out in our licence.

The Submitting Author accepts and understands that any supply made under these terms is made by BMJ to the Submitting Author unless you are acting as an employee on behalf of your employer or a postgraduate student of an affiliated institution which is paying any applicable article publishing charge ("APC") for Open Access articles. Where the Submitting Author wishes to make the Work available on an Open Access basis (and intends to pay the relevant APC), the terms of reuse of such Open Access shall be governed by a Creative Commons licence – details of these licences and which Creative Commons licence will apply to this Work are set out in our licence referred to above.



For peer review only - http://bmjopen.bmj.com/site/about/guidelines.xhtml

## References


1. LaRose JG, Gorin AA, Wing RR. Behavioral self-regulation for weight loss in young adults: a randomized controlled trial. Int J Behav Nutr Phy. 2009;6:10.
2. Oshima Y, Matsuoka Y, Sakane N. Effect of weight-loss program using self-weighing twice a day and feedback in overweight and obese subject: a randomized controlled trial. Obesity Research & Clinical Practice. 2013;7(5):e361-6.
3. Helander EE, Vuorinen AL, Wansink B, Korhonen IK. Are breaks in daily self-weighing associated with weight gain? PLoS One. 2014;9(11):e113164.
4. Steinberg DM, Tate DF, Bennett GG, Ennett S, Samuel-Hodge C, Ward DS. The efficacy of a daily self-weighing weight loss intervention using smart scales and e-mail. Obesity (Silver Spring). 2013;21(9):1789-97.
5. Burke LE, Wang J, Sevick MA. Self-monitoring in weight loss: a systematic review of the literature. Journal of The American Dietetic Association. 2011;111(1):92-102.
6. Kanfer FH. Self-Monitoring - Methodological Limitations and Clinical Applications. Journal of consulting and clinical psychology. 1970;35(2):148-52.
7. Boutelle K. Letters to the Editor. Journal of Nutrition Education and Behavior. 2006;38(3):131.
8. Kanfer FH, Karoly P. Self-Control: A Behavioristic Excursion into the Lion's Den. Behav Ther. 1972;3:398-416.
9. Hartmann-Boyce J, Boylan A, Jebb SA, Aveyard P. Experiences of self-monitoring in self-directed weight loss and weight loss maintenance: systematic review of qualitative studies. Qualitative Health Research. 2018:1049732318784815.
10. Daskalova N, Desingh K, Papoutsaki A, Schulze D, Sha H, Huang J. Lessons Learned from Two Cohorts of Personal Informatics Self-Experiments. Proceedings of the ACM on Interactive, Mobile, Wearable and Ubiquitous Technologies. 2017;1(3):46.
11. Madigan CD, Jolly K, Lewis AL, Aveyard P, Daley AJ. A randomised controlled trial of the effectiveness of self-weighing as a weight loss intervention. Int J Behav Nutr Phy. 2014;11:125.
12. Frie K, Hartmann-Boyce J, Jebb S, Pilbeam C, Aveyard P, editors. The role of self-regulation in and barriers to self-monitoring of weight. EHPS 2018; 2018 30.08.2018; Galway, Ireland: EHPS.
13. Pacanowski CR, Levitsky DA. Frequent Self-Weighing and Visual Feedback for Weight Loss in Overweight Adults. Journal of Obesity. 2015;2015:763680.
14. Leahey TM, Thomas G, Fava JL, Subak LL, Schembri M, Krupel K, et al. Adding evidence-based behavioral weight loss strategies to a statewide wellness campaign: a randomized clinical trial. American journal of public health. 2014;104(7):1300-6.
15. Bennett GG, Steinberg D, Askew S, Levine E, Foley P, Batch BC, et al. Effectiveness of an App and Provider Counseling for Obesity Treatment in Primary Care. American journal of preventive medicine. 2018;55(6):777-86.
16. Dombrowski SU, Endevelt R, Steinberg DM, Benyamini Y. Do more specific plans help you lose weight? Examining the relationship between plan




For peer review only - http://bmjopen.bmj.com/site/about/guidelines.xhtml

specificity, weight loss goals, and plan content in the context of a weight management programme. Br J Health Psychol. 2016;21(4):989-1005.
17. Benyamini Y, Geron R, Steinberg DM, Medini N, Valinsky L, Endevelt R. A structured intentions and action-planning intervention improves weight loss outcomes in a group weight loss program. American Journal of Health Promotion. 2013;28(2):119-27.
18. Lin M, Mahmooth Z, Dedhia N, Frutchey R, Mercado CE, Epstein DH, et al. Tailored, interactive text messages for enhancing weight loss among African American adults: the TRIMM randomized controlled trial. Am J Med. 2015;128(8):896-904.
19. Huisman S, de Gucht V, Maes S, Schroevers M, Chatrou M, Haak H. Self-regulation and weight reduction in patients with type 2 diabetes: a pilot intervention study. Patient Educ Couns. 2009;75(1):84-90.
20. Mann DM, Palmisano J, Lin JJ. A pilot randomized trial of technology-assisted goal setting to improve physical activity among primary care patients with prediabetes. Prev Med Rep. 2016;4:107-12.
21. Steinberg DM, Levine EL, Askew S, Foley P, Bennett GG. Daily text messaging for weight control among racial and ethnic minority women: randomized controlled pilot study. J Med Internet Res. 2013;15(11):e244.
22. Lin PH, Wang Y, Levine E, Askew S, Lin S, Chang C, et al. A text messaging-assisted randomized lifestyle weight loss clinical trial among overweight adults in Beijing. Obesity (Silver Spring). 2014;22(5):E29-37.
23. Huisman S, Maes S, De Gucht VJ, Chatrou M, Haak HR. Low goal ownership predicts drop-out from a weight intervention study in overweight patients with type 2 diabetes. Int J Behav Med. 2010;17(3):176-81.
24. Deci EL, Ryan RM. The "What" and "Why" of Goal Pursuits: Human Needs and the Self-Determination of Behavior. Psychological Inquiry. 2000;11(4):227-68.
25. Luszczynska A, Sobczyk A, Abraham C. Planning to lose weight: randomized controlled trial of an implementation intention prompt to enhance weight reduction among overweight and obese women. Health Psychology. 2007;26(4):507-12.
26. Linde JA, Jeffery RW, Crow SJ, Brelje KL, Pacanowski CR, Gavin KL, et al. The Tracking Study: description of a randomized controlled trial of variations on weight tracking frequency in a behavioral weight loss program. Contemp Clin Trials. 2015;40:199-211.
27. Morrison LG, Hargood C, Lin SX, Dennison L, Joseph J, Hughes S, et al. Understanding usage of a hybrid website and smartphone app for weight management: a mixed-methods study. J Med Internet Res. 2014;16(10):e201.
28. Lally P, Chipperfield A, Wardle J. Healthy habits: efficacy of simple advice on weight control based on a habit-formation model. International Journal of Obesity. 2008;32(4):700-7.
29. Madigan CD, Daley AJ, Lewis AL, Aveyard P, Jolly K. Is self-weighing an effective tool for weight loss: a systematic literature review and meta-analysis. Int J Behav Nutr Phy. 2015;12:104.
30. Burke LE, Conroy MB, Sereika SM, Elci OU, Styn MA, Acharya SD, et al. The effect of electronic self-monitoring on weight loss and dietary intake: a randomized behavioral weight loss trial. Obesity (Silver Spring). 2011;19(2):338-44.



For peer review only - http://bmjopen.bmj.com/site/about/guidelines.xhtml

31. Carter MC, Burley VJ, Nykjaer C, Cade JE. Adherence to a smartphone application for weight loss compared to website and paper diary: pilot randomized controlled trial. Journal of Medical Internet Research. 2013;15(4):e32.
32. Frie K, Hartmann-Boyce J, Jebb S, Albury C, Nourse R, Aveyard P. Insights From Google Play Store User Reviews for the Development of Weight Loss Apps: Mixed-Method Analysis. JMIR Mhealth Uhealth. 2017;5(12):e203.
33. de Vet E, Oenema A, Brug J. More or better: Do the number and specificity of implementation intentions matter in increasing physical activity? Psychology of Sport and Exercise. 2011;12(4):471-7.
34. Gollwitzer PM, Sheeran P. Implementation Intentions and Goal Achievement: A Meta‐analysis of Effects and Processes. Advances in Experimental Social Psychology Volume 38. Advances in Experimental Social Psychology. 382006. p. 69-119.
35. Hagger MS, Luszczynska A. Implementation intention and action planning interventions in health contexts: state of the research and proposals for the way forward. Applied Psychology: Health and Well-Being. 2014;6(1):1-47.
36. Hoffmann TC, Glasziou PP, Boutron I, Milne R, Perera R, Moher D, et al. Better reporting of interventions: template for intervention description and replication (TIDieR) checklist and guide. BMJ. 2014;348:g1687.
37. Vickers AJ, Altman DG. Statistics notes - Analysing controlled trials with baseline and follow up measurements. British Medical Journal. 2001;323(7321):1123-4.
38. Ritchie J, Spencer L. Qualitative Data Analysis for Applied Policy Research. In: Huberman A, Miles M, editors. The Qualitative Researcher's Companion. Thousand Oaks: SAGE Publications, Inc.; 2002. p. 305-29.
39. Braun V, Clarke V. Thematic Analysis. In: Cooper H, Camic, P. M., Long, D. L., Panter, A. T., Rindskopf, D. & Sher, K. J. , editor. APA handbook of research methods in psychology, Vol 2 Research designs: Quantitative, qualitative, neuropsychological, and biological. Washington, DC, US: American Psychological Association; 2012. p. 57-71.

For peer review only



For peer review only - http://bmjopen.bmj.com/site/about/guidelines.xhtml

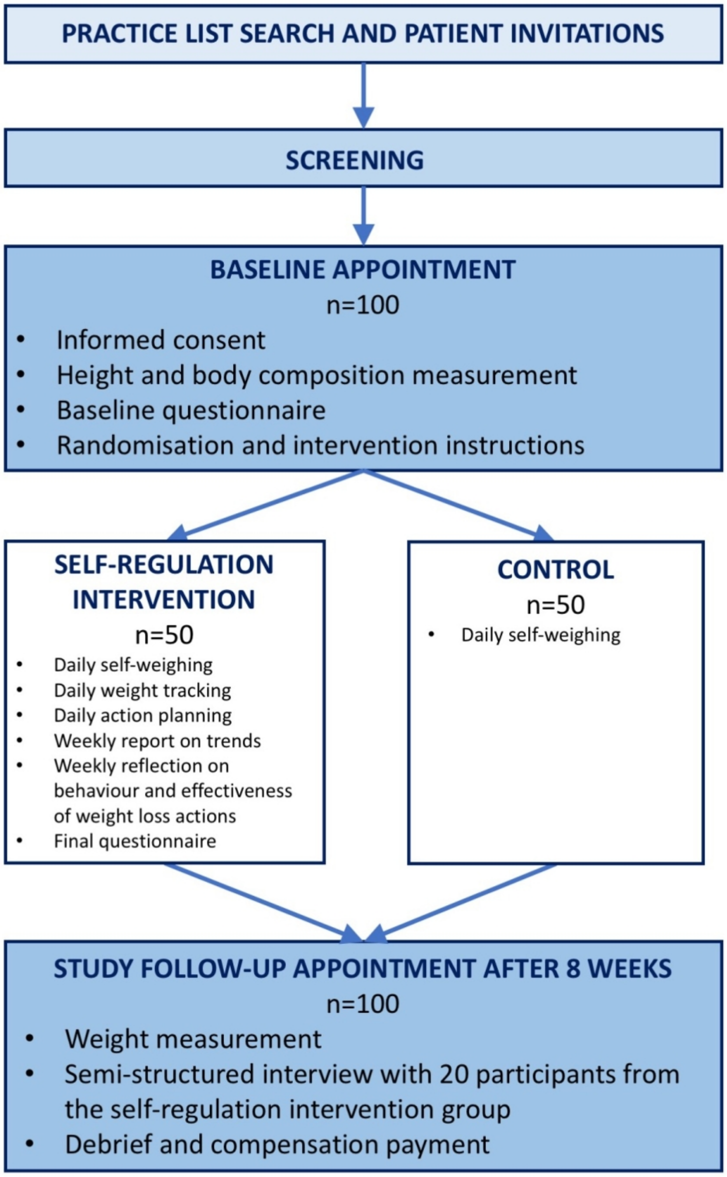


Study Flow Chart

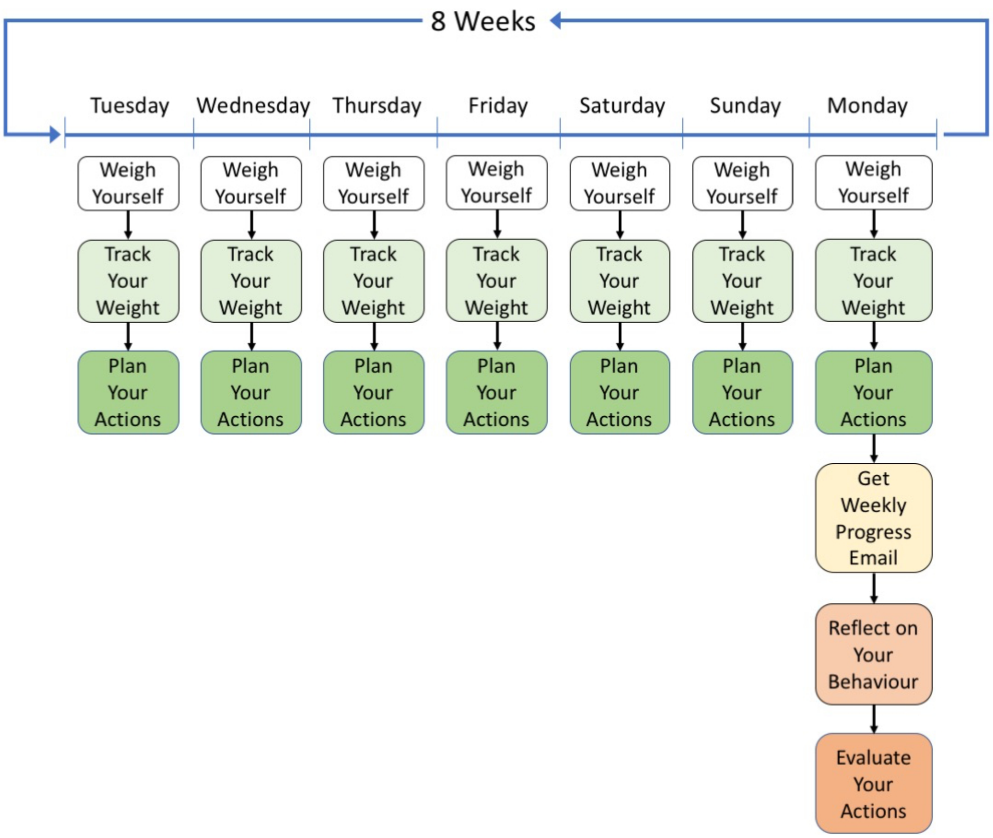


Intervention procedure as depicted in study manual

## List of 53 Weight Loss Actions

Category Red: Eating in a structured way
1. Plan all meals for the day in advance (what and when)
2. Eat no more than three times
3. Skip a meal
4. No calories after 8pm
5. Check the calorie count of everything you want to eat or drink
6. Set yourself a calorie goal and stick to it
7. Have a “fasting” day with less than 800kcal
8. Keep a diary of what you eat and how you feel
9. Check your portion size
10. Only eat when sitting at a table

Category Orange: Avoiding or swapping specific foods
1. Don’t eat between meals
2. Cut out crisps, biscuits, cakes and sweets
3. Cut out fried food
4. Have only one course at meal-times
5. Cut out carbs
6. Swap unhealthy snacks for fruits and vegetables
7. Swap rice/potatoes/pasta for extra vegetables
8. Use meal replacement products
9. Swap unhealthy snacks with 6-8 individual nuts

Category Yellow: Changing what you drink
1. Drink only water or unsweetened coffee or tea
2. Swap sugary soft drinks with diet or no sugar versions
3. Do not drink alcohol
4. Drink a pint of water before each meal
5. Swap juices or smoothies with whole fruit and vegetables

Category Green: Creating a healthier diet
1. Eat at least 5 portions of fruit or vegetables each day
2. Snack only on vegetables
3. Eat only foods with a green nutrition label for total fat
4. Eat only foods with a green nutrition label for sugar
5. Make sure half of your main meal of the day is a salad or vegetables
6. Swap rice/potatoes/pasta with extra vegetables
7. Swap fatty meats with lean meats



For peer review only - http://bmjopen.bmj.com/site/about/guidelines.xhtml

Category Blue: Meal-time tactics

1. Eat slowly or 20 chews per bite
2. Focus on your food while eating
3. Stop eating before you feel full
4. Use smaller plates and bowls
5. Cut food into smaller pieces
6. Eat for less than 20 minutes at a time

Category Purple: Burn more calories

1. Walk up and down a flight of stairs for as long as you can
2. Go cycling for as long as you can
3. Go swimming for as long as you can
4. Stretching Exercises
5. Attend an exercise class
6. Play a group sport
7. Go to the gym
8. Exercise at home with the 21-minutes NHS Choices workout
9. Brisk walking for as long as you can

Category Pink: Be more active as part of your daily life

1. Walk 10,000 steps
2. Walk/cycle instead of taking the bus or car
3. Go for a walk with your friend(s)
4. Stand up while working
5. Take the stairs whenever you can
6. Have an active day with your family or friends
7. Stand up while watching TV



For peer review only - http://bmjopen.bmj.com/site/about/guidelines.xhtml

# STATISTICAL ANALYSIS PLAN

**Study Title:**
A proof of concept trial of a self-regulation intervention for weight loss
(PREVAIL Trial)



| | |
|---|---|
| **Chief Investigator:** | Kerstin Frie, Doctoral Candidate,<br>Nuffield Department of Primary Care Health Sciences |
| **Co-Investigators:** | Dr Jamie Hartmann-Boyce[1]<br>Prof. Susan Jebb[1]<br>Prof. Paul Aveyard[1]<br>1. Nuffield Department of Primary Care Health Sciences |



<u>Version History</u>

| Version: | Version Date: | Changes: |
|---|---|---|
| 1.0 | **21st October 2019** | |



For peer review only - http://bmjopen.bmj.com/site/about/guidelines.xhtml

Table of Contents

**INTRODUCTION** ..... **3**

**PREFACE** ..... 3
**PURPOSE AND SCOPE OF THE STATISTICAL ANALYSIS PLAN** ..... 3
**BACKGROUND** ..... 3
**TRIAL OVERVIEW** ..... 3
**OBJECTIVES** ..... 4
PRIMARY OUTCOME ..... 4
SECONDARY OUTCOMES (PROCESS EVALUATION) ..... 4

**TRIAL DESIGN** ..... **4**

**OUTCOME MEASURES** ..... 4
**PRIMARY OUTCOME** ..... 4
**SECONDARY OUTCOMES (PROCESS EVALUATION)** ..... 5
**BLINDING IN THE ANALYSIS STAGE** ..... 6

**PRIMARY & SECONDARY ANALYSIS** ..... **6**

**PRIMARY OUTCOME** ..... 6
**SECONDARY OUTCOMES (PROCESS EVALUATION)** ..... 6

**ANALYSIS – GENERAL CONSIDERATIONS** ..... **7**

**DATA CLEANING** ..... 7
**DESCRIPTIVE STATISTICS AND PARTICIPANT CHARACTERISTICS** ..... 7
**DEFINITION OF POPULATION FOR ANALYSIS** ..... 7
**HANDLING MISSING DATA** ..... 8
**HANDLING OUTLIERS** ..... 8
**MODEL ASSUMPTIONS** ..... 8

**APPENDICES** ..... **9**

For peer review only



For peer review only - http://bmjopen.bmj.com/site/about/guidelines.xhtml

# Introduction

## Preface

The SAP supports the study protocol version 1.1, dated 07/12/2018. Quantitative analysis will be carried out using SPSS, version 24, and R Studio, version 1.0.153. Qualitative analysis will be run using NVivo, version 11.4.2.

## Purpose and scope of the statistical analysis plan

The purpose of the plan is to set out the main analysis as stated in the protocol.

## Background

The effectiveness of self-weighing for weight loss has often been ascribed to an automatic self-regulation mechanism. It starts with the contextualisation of weight measurements, thus providing an opportunity to reflect on previous behaviours, and enabling the planning and performance of weight loss actions. A study employing self-weighing as a standalone intervention did not find a significant weight loss effect, raising the question whether self-regulation is performed naturally after weighing. We addressed this question in a think-aloud study, where twenty-four participants were asked to record their thoughts and feelings during daily weighing for eight weeks, without being prompted to self-regulate. On 90% of occasions, participants contextualised their weight measurements and on 58% participants reflected on previous behaviours. Only on 20% of occasions did participants plan actions. Specific action-planning, defining a concrete action and time plan, was rare (6%). The frequency of specific action-planning was, however, significantly predictive of weight loss. Hence, the study provided support to the notion that completing the last step of the self-regulation process can elicit weight loss. However, the think-aloud study also showed that self-regulation does not often occur autonomously, and that people need support in developing self-regulation skills, especially action-planning.

## Trial Overview

With this study we aim to test the early effectiveness of an intervention guiding people through the iterative self-regulation process. One hundred participants with a BMI≥30 kg/m2 will be randomised to either the control or intervention group. The control group will be asked to weigh themselves daily for eight weeks, the intervention group will be encouraged to follow the self-regulation intervention. They will be prompted to weigh daily, track their weight using an app, plan daily actions for weight loss and reflect on their action plans on a weekly basis. This self-regulation cycle will allow them to experiment with different weight loss strategies and identify effective and sustainable actions. Primary and process outcomes will be measured at baseline and 8-weeks follow-up.



For peer review only - http://bmjopen.bmj.com/site/about/guidelines.xhtml

## Objectives

### Primary Outcome

The primary objective of this trial is to test the concept of whether an intervention, which trains individuals in self-regulatory processes following self-weighing, increases early weight loss in comparison to unsupported daily weighing.

### Secondary Outcomes (Process Evaluation)

A mix of quantitative and qualitative measures will be used to undertake a process evaluation of the intervention and investigate moderators of effectiveness. This will include a quantitative assessment of participants' adherence to intervention components. The resulting measures will be used to assess whether adherence is predictive of weight change. Furthermore, as cognitive processing and reflection skills are required for self-regulation, the predictive value of the highest educational qualification achieved will be assessed for the outcome weight change. Since weighing is a major component of the treatment in both the control and intervention group, a potential link between liking of weighing at baseline and weight change at follow-up will also be investigated. Furthermore, the perceived usefulness of intervention features shall be explored quantitatively. The association between the overall intervention rating and weight change from baseline to follow-up will be assessed. Qualitative analysis of semi-structured interviews will help to evaluate acceptability and feasibility of the intervention, as well as identify barriers and unmet needs.

## Trial design

An individually randomised, two arm, parallel group design, assessing superiority of the self-regulation intervention over daily self-weighing alone. Participation lasts eight weeks and participants are randomly allocated to the intervention or control condition on a 1:1 basis, stratified by GP practice. The intervention features are detailed in the study protocol. The study population includes 100 adults ≥18 years of age, with a BMI≥30 kg/$m^2$, who did not weigh themselves more than once a week at time of enrolment. For further details, refer to the trial protocol.

## Outcome measures

### Primary outcome

| Primary Objective | Measures | Timepoints |
| --- | --- | --- |
| **Early effectiveness** | Change in weight from baseline to follow-up, using a digital scale. | Baseline to follow-up (8-9 weeks) |



For peer review only - http://bmjopen.bmj.com/site/about/guidelines.xhtml

## Secondary outcomes (Process Evaluation)

| Objectives | Outcome Measures | Timepoints of evaluation of this outcome measure |
|---|---|---|
| **1. Adherence** | Intervention Condition: Adherence to weight-tracking will be measured as the proportion of days for which a weight was recorded in the weight-tracking app. Adherence to action planning and reflection/evaluation will be measured by calculating the proportion of days/weeks on which the respective questionnaires were completed. The proportionate adherence to daily weighing will be pooled from weight-tracking and action planning questionnaire data. A composite adherence score will be calculated by averaging the adherence measures for daily weighing, weight-tracking, action planning and reflection/evaluation.<br>Control Condition: Adherence to daily weighing will be measured as the proportion of days for which a weight was recorded on the BodyTrace scales server or for which a written record was provided by the participant. | Throughout intervention period |
| **2. Baseline Questionnaire** | A baseline questionnaire measures demographic characteristics such age, gender, ethnicity, highest educational degree and employment status. The questionnaire further asks participants whether they like weighing themselves on a scale from 1 (dislike it a great deal) to 5 (like it a great deal). | At baseline |
| **3. Final Questionnaire** | A final questionnaire collects ratings on a scale from 1 (not useful) to 10 (very useful) for each intervention component, as well as for the intervention overall, from participants in the intervention condition. | At follow-up |
| **3. Semi-structured Interviews** | Semi-structured interviews are conducted by the researcher at follow-up in order to gain insights into the participants' opinions regarding the intervention components, as well as to collect feedback on acceptability and feasibility, alongside identifying barriers and unmet needs. | At follow-up |



For peer review only - http://bmjopen.bmj.com/site/about/guidelines.xhtml

### BLINDING IN THE ANALYSIS STAGE

It is not possible to blind the researcher delivering the intervention and analysing the trial data to treatment group due to the nature of the intervention. The primary outcome, change in weight, is measured objectively and is therefore unlikely to be biased. Adherence to self-regulation steps is also measured objectively through the frequency of weight logs and completed questionnaires in control and intervention group. The evaluations of treatment components are measured in an online questionnaire without the researchers input. Blinding of the researcher who conducts and analyses the semi-structured interviews with intervention group participants will not be possible.

## PRIMARY & SECONDARY ANALYSIS

### PRIMARY OUTCOME

The statistical analysis of the primary outcome, effectiveness of the intervention for weight loss, will be carried out both on the basis of intention-to-treat (ITT) and per-protocol (PP). For the ITT analysis, participants will be analysed according to their allocated intervention group. We will endeavour to obtain full follow-up data on every participant to allow full ITT analysis. Where we are unable to meet participants for follow-up, we will try to record self-reported weight at eight-week follow-up by telephone or email. For the PP analysis, we will exclude participants who stopped following their allocated intervention at some point throughout the study. In both cases, a linear mixed effects model, predicting weight at follow-up while adjusting for baseline weight (fixed effect) and GP practice (random effect), will assess the effect of condition (fixed effect). All analysis will be done at a 5% two-sided significance level.

### SECONDARY OUTCOMES (PROCESS EVALUATION)

Means and standard deviations of adherence rates to the different intervention components will be calculated. Adherence rates for daily weighing in the control and intervention condition will be compared using independent samples t-tests. Means and standard deviations of final questionnaire ratings will be calculated.

Further linear mixed effects models will be calculated to assess different potential moderators of effectiveness.

Highest educational qualification: Since the majority of participants in the sample have a university degree or equivalent, and the rest are distributed in small quantities across the other qualification levels, a binary variable with 1=university degree or equivalent and 0=no university degree or equivalent will be calculated. A linear mixed effects model using the same parameters as in the primary analysis, and adding the binary variable educational qualification, as well as the interaction term education*condition, will be run in order to test



For peer review only - http://bmjopen.bmj.com/site/about/guidelines.xhtml

for an effect on weight change.

Adherence: We will replicate the linear mixed effects model of the primary analysis, and add the composite adherence score in the intervention group/the daily weighing adherence measure in the control condition in order to assess the predictive value of overall adherence for weight change. The model will also include an interaction term condition*adherence.

Liking of Weighing: A linear mixed effects model will test the predictive value of liking of weighing at baseline for weight change, adjusting for the same parameters as in the primary analysis. Liking of weighing will be added both as an independent parameter, as well as in an interaction term with condition.

Intervention Rating (only intervention condition): A linear mixed effects model will test the predictive value of the overall intervention rating for weight change, adjusting for both baseline weight and GP practice.

All of the above analyses will be done at a 5% two-sided significance level.

All interview audio-recordings will be transcribed and entered into the NVivo software package (QSR International) for qualitative data analysis. Framework analysis according to Ritchie and Spencer will assess the participant's experiences and perceptions of the different intervention components. The findings will be put into context with the results from the final questionnaire. Inductive thematic analysis following Braun and Clarke will explore additional themes, including acceptability, barriers and unmet needs.

# Analysis – General considerations

## Data cleaning

Prior to the final data lock, data cleaning will be performed, including checking that all appropriate data has been reported.

## Descriptive statistics and participant characteristics

A table will present the baseline characteristics by trial arm and overall (Appendix 1).

Continuous variables will be summarised using means and standard deviations. Categorical variables will be summarised using counts and percentages. Data will be analysed using R.

## Definition of population for analysis

The statistical analysis of efficacy outcomes will be carried out on the basis of intention-to-treat (ITT). We will endeavour to obtain full follow-up data on every participant to allow full ITT analysis, but we will inevitably experience the problem of missing data due to withdrawal, loss to follow up, or non-response to questionnaire items.



For peer review only - http://bmjopen.bmj.com/site/about/guidelines.xhtml

## Handling missing data

The percentage and absolute withdrawal of participants lost-to-follow up will be reported for each study arm in the CONSORT flow-chart and reasons for missing data will be documented.

Primary analysis:

Where it is not possible to obtain full follow-up data for the primary outcome, baseline observations will be carried forward. We will assess the sensitivity of the analysis to assumptions about missing data by also running an analysis restricted to participants completing follow-up and an analysis imputing the last home-measured weight for people who did not attend the final meeting and did not self-report their weight at eight-week follow-up. All tests will be done at a 5% two-sided significance level.

Secondary analyses:

If participants in the control condition experience issues with the synchronisation of weight measurements to the BodyTrace server, self-reported written records of weighing data will be accepted as measures of weighing adherence. Where control group participants do not keep a written record of measurements, the proportion of days adherent to weighing before the issues with synchronisation arose will be calculated.
Where participants in the intervention condition do not use the weight-tracking app or the app data is faulty, self-reported written records of weight measurements will be accepted as weight-tracking adherence measures. Where the weight-tracking data is incomplete due to technical reasons (e.g. switching of mobile phones), the proportion of days adherent to weight-tracking will be calculated for the time frame covered by the existing data. Missing data will not be imputed for the moderator variables. That is, cases with missing data on adherence and intervention rating will be excluded from the moderator analyses.

## Handling outliers

We do not expect significant outliers based on our definition of population for analysis.

## Model assumptions

For the primary analysis, the normality of all model residuals will be assessed using histograms, QQ-plots and other diagnostic plots. Where the normality assumption is violated, a sensitivity analysis using semi-parametric generalized estimating equations will be run additionally.



For peer review only - http://bmjopen.bmj.com/site/about/guidelines.xhtml

# APPENDICES

***Appendix 1. Template tables for presentation of results***

**Baseline Demographic Characteristics**

| N(%), unless otherwise specified | Control (n=) | Intervention (n=) | Total (n=) |
|---|---|---|---|
| Age, years, mean (SD) | | | |
| Gender, % female | | | |
| BMI, kg/m$^2$, mean (SD) | | | |
| Ethnicity | | | |
| White | | | |
| Asian or Asian British | | | |
| Black of Black British | | | |
| Mixed/Other | | | |
| Highest Educational Qualification | | | |
| No formal qualifications | | | |
| Vocational/work-related qualifications | | | |
| GCSE, NVQ level 1 | | | |
| Apprenticeship | | | |
| A' levels, NVQ level 2-3 | | | |
| Other post-high school qualifications | | | |
| University Degree, NVQ level 4+ | | | |
| Employment Status | | | |
| Employed | | | |
| Self-employed | | | |
| Unemployed | | | |
| Looking after home and family | | | |
| Student | | | |
| Retired | | | |
| Long-term sick or disabled | | | |



For peer review only - http://bmjopen.bmj.com/site/about/guidelines.xhtml

For peer review only

**Other Baseline Characteristics**

| N(%), unless otherwise specified | Control (n=) | Intervention (n=) | Total (n=) |
|---|---|---|---|
| Weighing Frequency | | | |
| Less than once a month | | | |
| Once a month | | | |
| Every other week | | | |
| Once a week | | | |
| Liking of weighing | | | |
| Dislike it a great deal | | | |
| Dislike it somewhat | | | |
| Neither like nor dislike it | | | |
| Like it somewhat | | | |
| Like it a great deal | | | |
| Usefulness of weighing to control weight | | | |
| Definitely not | | | |
| Probably not | | | |
| I don't know | | | |
| Probably yes | | | |
| Definitely yes | | | |

For peer review only



For peer review only - http://bmjopen.bmj.com/site/about/guidelines.xhtml

NUFFIELD DEPARTMENT OF PRIMARY CARE HEALTH SCIENCES

UNIVERSITY OF OXFORD

## Primary Outcome (Effectiveness)

### Linear Mixed Effects Model

| | | Mean (SD) weight change from baseline | | | |
|---|---|---|---|---|---|
| | | Intervention | Control | Adjusted difference (95% CI) | *p* |
| Intention to Treat Analysis | Baseline Observation Carried Forward (N=) | | | | |
| | Last Home-measured Weight (N=) | | | | |
| | Completed Follow-up (N=) | | | | |
| Per Protocol Analysis (N=) | | | | | |
| *Adjusted for: GP practice (random effect), baseline weight (fixed effect)* | | | | | |

For peer review only

The Nuffield Department of Primary Care Health Sciences is part of the NIHR School for Primary Care Research. Head of Department: Professor Richard Hobbs FRCGP FRCP FESC FMedSci

Athena SWAN Silver Award

UNIVERSITY OF OXFORD
NUFFIELD DEPARTMENT OF PRIMARY CARE HEALTH SCIENCES

## Secondary Outcomes (Process Evaluation)

### Adherence Rates

| Intervention Component | Mean % (SD) |
|---|---|
| *Control* | |
| Daily Weighing (n=) | |
| *Intervention* | |
| Daily Weighing (n=) | |
| Weight-Tracking (n=) | |
| Daily Action Planning Questionnaires (n=) | |
| Weekly Reflection/Evaluation Questionnaires (n=) | |
| Action Diary (optional, n=) | |

### Adherence Rates Control vs Intervention

| **Control vs Intervention** | df | *t* | *p* | 95% CI |
|---|---|---|---|---|
| Daily Weighing (N=) | | | | |

For peer review only

NUFFIELD DEPARTMENT OF PRIMARY CARE HEALTH SCIENCES

**Intervention Component Rating (only intervention condition)**

| Question | Mean (SD) |
|---|---|
| 1. How do you feel about weighing yourself overall? (n=) | |
| 2. How useful did you find the intervention for controlling your weight overall? (n=) | |
| 3. How useful did you find tracking your weight for controlling your weight? (n=) | |
| 4. How useful did you find planning weight loss actions for controlling your weight? (n=) | |
| 5. How useful did you find reflecting on the reasons for weight changes for controlling your weight? (n=) | |
| 6. How useful did you find the weekly action evaluation for controlling your weight? (n=) | |

Questions were completed by members of the intervention condition who attended the follow-up meeting. Question 1 was rated on a scale from 1 (very negative) to 10 (very positive). Questions 2-6 were rated on a scale from 1 (not useful) to 10 (very useful).

The Nuffield Department of Primary Care Health Sciences is part of the NIHR School for Primary Care Research. Head of Department: Professor Richard Hobbs FRCGP FRCP FESC FMedSci

Athena SWAN Silver Award

NUFFIELD DEPARTMENT OF PRIMARY CARE HEALTH SCIENCES
UNIVERSITY OF OXFORD

## Moderators of Effectiveness

## Linear Mixed Effects Models

| | | Adjusted Difference (95% CI); p | | |
|---|---|---|---|---|
| | | Condition | Additional Variable | Additional Variable*Condition |
| Moderator Variables | Adherence (N=) | | | |
| | University degree (N=) | | | |
| | Liking of weighing (N=) | | | |
| | Overall intervention rating (N=) | - | | - |
| Outcome: Weight Change (BOCF); adjusted for: GP practice (random effect), baseline weight (fixed effect) | | | | |

For peer review only

SPIRIT
STANDARD PROTOCOL ITEMS: RECOMMENDATIONS FOR INTERVENTIONAL TRIALS

SPIRIT 2013 Checklist: Recommended items to address in a clinical trial protocol and related documents*

| Section/item | Item No | Description | |
|---|---|---|---|
| **Administrative information** | | | |
| Title | 1 | Descriptive title identifying the study design, population, interventions, and, if applicable, trial acronym | ☑ p. 1 |
| Trial registration | 2a | Trial identifier and registry name. If not yet registered, name of intended registry | ☑ p. 2 |
| | 2b | All items from the World Health Organization Trial Registration Data Set | ☑ p. 1-17 |
| Protocol version | 3 | Date and version identifier | ☑ p. 2 |
| Funding | 4 | Sources and types of financial, material, and other support | ☑ p. 17 |
| Roles and responsibilities | 5a | Names, affiliations, and roles of protocol contributors | ☑ p. 1, 17 |
| | 5b | Name and contact information for the trial sponsor | ☑ p. 17 |
| | 5c | Role of study sponsor and funders, if any, in study design; collection, management, analysis, and interpretation of data; writing of the report; and the decision to submit the report for publication, including whether they will have ultimate authority over any of these activities | ☑ p. 17 |
| | 5d | Composition, roles, and responsibilities of the coordinating centre, steering committee, endpoint adjudication committee, data management team, and other individuals or groups overseeing the trial, if applicable (see Item 21a for data monitoring committee) | ☑ p. 15-16 |
| **Introduction** | | | |
| Background and rationale | 6a | Description of research question and justification for undertaking the trial, including summary of relevant studies (published and unpublished) examining benefits and harms for each intervention | ☑ p. 4-5 |

For peer review only

| | | | |
|---|---|---|---|
| | 6b | Explanation for choice of comparators | ☑ p. 8-12 |
| Objectives | 7 | Specific objectives or hypotheses | ☑ p. 5 |
| Trial design | 8 | Description of trial design including type of trial (eg, parallel group, crossover, factorial, single group), allocation ratio, and framework (eg, superiority, equivalence, noninferiority, exploratory) | ☑ p. 5 |

**Methods: Participants, interventions, and outcomes**

| | | | |
|---|---|---|---|
| Study setting | 9 | Description of study settings (eg, community clinic, academic hospital) and list of countries where data will be collected. Reference to where list of study sites can be obtained | ☑ p. 5 |
| Eligibility criteria | 10 | Inclusion and exclusion criteria for participants. If applicable, eligibility criteria for study centres and individuals who will perform the interventions (eg, surgeons, psychotherapists) | ☑ p. 6 |
| Interventions | 11a | Interventions for each group with sufficient detail to allow replication, including how and when they will be administered | ☑ p. 8-12 |
| | 11b | Criteria for discontinuing or modifying allocated interventions for a given trial participant (eg, drug dose change in response to harms, participant request, or improving/worsening disease) | ☑ p. 15 |
| | 11c | Strategies to improve adherence to intervention protocols, and any procedures for monitoring adherence (eg, drug tablet return, laboratory tests) | ☑ p. 13-14 |
| | 11d | Relevant concomitant care and interventions that are permitted or prohibited during the trial | ☑ p. 6 |
| Outcomes | 12 | Primary, secondary, and other outcomes, including the specific measurement variable (eg, systolic blood pressure), analysis metric (eg, change from baseline, final value, time to event), method of aggregation (eg, median, proportion), and time point for each outcome. Explanation of the clinical relevance of chosen efficacy and harm outcomes is strongly recommended | ☑ p. 12-13 |
| Participant timeline | 13 | Time schedule of enrolment, interventions (including any run-ins and washouts), assessments, and visits for participants. A schematic diagram is highly recommended (see Figure) | ☑ p. 6-7, 14 |
| Sample size | 14 | Estimated number of participants needed to achieve study objectives and how it was determined, including clinical and statistical assumptions supporting any sample size calculations | ☑ p. 7 |
| Recruitment | 15 | Strategies for achieving adequate participant enrolment to reach target sample size | ☑ p. 5-6 |



For peer review only - http://bmjopen.bmj.com/site/about/guidelines.xhtml

**Methods: Assignment of interventions (for controlled trials)**

Allocation:

| | | | |
|---|---|---|---|
| Sequence generation | 16a | Method of generating the allocation sequence (eg, computer-generated random numbers), and list of any factors for stratification. To reduce predictability of a random sequence, details of any planned restriction (eg, blocking) should be provided in a separate document that is unavailable to those who enrol participants or assign interventions | ☑ p. 8 |
| Allocation concealment mechanism | 16b | Mechanism of implementing the allocation sequence (eg, central telephone; sequentially numbered, opaque, sealed envelopes), describing any steps to conceal the sequence until interventions are assigned | ☑ p. 8 |
| Implementation | 16c | Who will generate the allocation sequence, who will enrol participants, and who will assign participants to interventions | ☑ p. 8 |
| Blinding (masking) | 17a | Who will be blinded after assignment to interventions (eg, trial participants, care providers, outcome assessors, data analysts), and how | ☑ p. 8 |
| | 17b | If blinded, circumstances under which unblinding is permissible, and procedure for revealing a participant's allocated intervention during the trial | N/A |

**Methods: Data collection, management, and analysis**

| | | | |
|---|---|---|---|
| Data collection methods | 18a | Plans for assessment and collection of outcome, baseline, and other trial data, including any related processes to promote data quality (eg, duplicate measurements, training of assessors) and a description of study instruments (eg, questionnaires, laboratory tests) along with their reliability and validity, if known. Reference to where data collection forms can be found, if not in the protocol | ☑ p. 12-13 |
| | 18b | Plans to promote participant retention and complete follow-up, including list of any outcome data to be collected for participants who discontinue or deviate from intervention protocols | ☑ p. 14 |
| Data management | 19 | Plans for data entry, coding, security, and storage, including any related processes to promote data quality (eg, double data entry; range checks for data values). Reference to where details of data management procedures can be found, if not in the protocol | ☑ p. 16 |
| Statistical methods | 20a | Statistical methods for analysing primary and secondary outcomes. Reference to where other details of the statistical analysis plan can be found, if not in the protocol | ☑ p. 14-15 |

| | | | |
|---|---|---|---|
| | 20b | Methods for any additional analyses (eg, subgroup and adjusted analyses) | ☑ p. 14-15 |
| | 20c | Definition of analysis population relating to protocol non-adherence (eg, as randomised analysis), and any statistical methods to handle missing data (eg, multiple imputation) | ☑ p. 14-15 |
| **Methods: Monitoring** | | | |
| Data monitoring | 21a | Composition of data monitoring committee (DMC); summary of its role and reporting structure; statement of whether it is independent from the sponsor and competing interests; and reference to where further details about its charter can be found, if not in the protocol. Alternatively, an explanation of why a DMC is not needed | ☑ p. 16 |
| | 21b | Description of any interim analyses and stopping guidelines, including who will have access to these interim results and make the final decision to terminate the trial | N/A |
| Harms | 22 | Plans for collecting, assessing, reporting, and managing solicited and spontaneously reported adverse events and other unintended effects of trial interventions or trial conduct | ☑ p. 15 |
| Auditing | 23 | Frequency and procedures for auditing trial conduct, if any, and whether the process will be independent from investigators and the sponsor | ☑ p. 16 |
| **Ethics and dissemination** | | | |
| Research ethics approval | 24 | Plans for seeking research ethics committee/institutional review board (REC/IRB) approval | ☑ p. 16 |
| Protocol amendments | 25 | Plans for communicating important protocol modifications (eg, changes to eligibility criteria, outcomes, analyses) to relevant parties (eg, investigators, REC/IRBs, trial participants, trial registries, journals, regulators) | ☑ p. 16 |
| Consent or assent | 26a | Who will obtain informed consent or assent from potential trial participants or authorised surrogates, and how (see Item 32) | ☑ p. 7 |
| | 26b | Additional consent provisions for collection and use of participant data and biological specimens in ancillary studies, if applicable | N/A |
| Confidentiality | 27 | How personal information about potential and enrolled participants will be collected, shared, and maintained in order to protect confidentiality before, during, and after the trial | ☑ p. 16 |
| Declaration of interests | 28 | Financial and other competing interests for principal investigators for the overall trial and each study site | ☑ p. 17 |

For peer review only

| | | | |
|---|---|---|---|
| Access to data | 29 | Statement of who will have access to the final trial dataset, and disclosure of contractual agreements that limit such access for investigators | ☑ p. 16 |
| Ancillary and post-trial care | 30 | Provisions, if any, for ancillary and post-trial care, and for compensation to those who suffer harm from trial participation | N/A |
| Dissemination policy | 31a | Plans for investigators and sponsor to communicate trial results to participants, healthcare professionals, the public, and other relevant groups (eg, via publication, reporting in results databases, or other data sharing arrangements), including any publication restrictions | ☑ p. 16 |
| | 31b | Authorship eligibility guidelines and any intended use of professional writers | ☑ p. 16 |
| | 31c | Plans, if any, for granting public access to the full protocol, participant-level dataset, and statistical code | N/A |
| **Appendices** | | | |
| Informed consent materials | 32 | Model consent form and other related documentation given to participants and authorised surrogates | ☑ |
| Biological specimens | 33 | Plans for collection, laboratory evaluation, and storage of biological specimens for genetic or molecular analysis in the current trial and for future use in ancillary studies, if applicable | N/A |

*It is strongly recommended that this checklist be read in conjunction with the SPIRIT 2013 Explanation & Elaboration for important clarification on the items. Amendments to the protocol should be tracked and dated. The SPIRIT checklist is copyrighted by the SPIRIT Group under the Creative Commons "Attribution-NonCommercial-NoDerivs 3.0 Unported" license.

For peer review only
